# Multivariate Bayesian Optimization of CoO Nanoparticles for CO_2_ Hydrogenation Catalysis

**DOI:** 10.1021/jacs.4c03789

**Published:** 2024-05-10

**Authors:** Lanja R. Karadaghi, Emily M. Williamson, Anh T. To, Allison P. Forsberg, Kyle D. Crans, Craig L. Perkins, Steven C. Hayden, Nicole J. LiBretto, Frederick G. Baddour, Daniel A. Ruddy, Noah Malmstadt, Susan E. Habas, Richard L. Brutchey

**Affiliations:** † Department of Chemistry, University of Southern California, Los Angeles, California 90089, United States; ‡ Catalytic Carbon Transformation and Scale-Up Center, National Renewable Energy Laboratory, Golden, Colorado 80401, United States; § Materials Science Center, National Renewable Energy Laboratory, Golden, Colorado 80401, United States; ∥ Mork Family Department of Chemical Engineering and Materials Science, University of Southern California, Los Angeles, California 90089, United States; ⊥ Department of Biomedical Engineering, University of Southern California, Los Angeles, California 90089, United States; # USC Norris Comprehensive Cancer Center, University of Southern California, 1441 Eastlake Avenue, Los Angeles, California 90033, United States

## Abstract

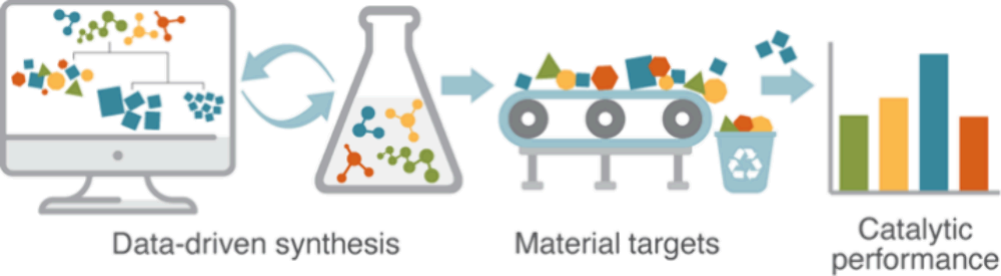

The hydrogenation of CO_2_ holds promise for transforming the production of renewable fuels and chemicals. However, the challenge lies in developing robust and selective catalysts for this process. Transition metal oxide catalysts, particularly cobalt oxide, have shown potential for CO_2_ hydrogenation, with performance heavily reliant on crystal phase and morphology. Achieving precise control over these catalyst attributes through colloidal nanoparticle synthesis could pave the way for catalyst and process advancement. Yet, navigating the complexities of colloidal nanoparticle syntheses, governed by numerous input variables, poses a significant challenge in systematically controlling resultant catalyst features. We present a multivariate Bayesian optimization, coupled with a data-driven classifier, to map the synthetic design space for colloidal CoO nanoparticles and simultaneously optimize them for multiple catalytically relevant features within a target crystalline phase. The optimized experimental conditions yielded small, phase-pure rock salt CoO nanoparticles of uniform size and shape. These optimized nanoparticles were then supported on SiO_2_ and assessed for thermocatalytic CO_2_ hydrogenation against larger, polydisperse CoO nanoparticles on SiO_2_ and a conventionally prepared catalyst. The optimized CoO/SiO_2_ catalyst consistently exhibited higher activity and CH_4_ selectivity (ca. 98%) across various pretreatment reduction temperatures as compared to the other catalysts. This remarkable performance was attributed to particle stability and consistent H* surface coverage, even after undergoing the highest temperature reduction, achieving a more stable catalytic species that resists sintering and carbon occlusion.

## Introduction

The selective hydrogenation of CO_2_ to value-added chemicals and fuels not only reduces our dependence on nonrenewable resources but also has the added benefit of utilizing waste CO_2_ instead of emitting it to the atmosphere.^1−3^ A variety of catalysts for thermocatlytic CO_2_ hydrogenation including metals,^4,5^ intermetallic compounds,^6,7^ and metal oxides^8−10^ have been developed for reverse water gas shift, methanol synthesis, methanation, and C–C coupling reactions to yield long-chain alkanes and higher alcohols.^11−13^ One highly studied class of materials for CO_2_ hydrogenation catalysis is transition metal oxides. Among these, cobalt oxide is a transition metal oxide of interest because of its versatility across a broad range of catalytic transformations, including electrochemical water splitting, CO oxidation, and nitric oxide reduction, as well as CO_2_ hydrogenation.^14−16^ However, the complexity of cobalt oxide-based catalysts, which can include multiple valence states (e.g., Co^2+^ and Co^3+^) and crystal structures, coupled with the variety of transformations they can promote, has led to challenges in understanding and tailoring robust and selective catalysts.^17−21^ Two of the most thermodynamically stable phases of cobalt oxide are CoO, which has the rock salt structure type, and Co_3_O_4_, which has the spinel structure type. Spinel Co_3_O_4_ is a mixed-valent compound, with Co^3+^ formally residing in the octahedral sites and Co^2+^ formally residing in tetrahedral sites, while rock salt CoO exclusively contains Co^2+^ in octahedral sites. In addition to crystal phase, the morphology of cobalt oxide-based catalysts has also been shown to impact CO_2_ hydrogenation, although gaining synthetic control over these features with the goal of promoting catalytic selectivity is still very much a challenge.^17−21^ Recent efforts have focused on tailoring the crystal phase and morphology of nanostructured catalysts through solution synthesis routes, enabling the development of structure-performance relationships that can drive advances in catalytic materials.^22,23^

Attaining precise morphological control (i.e., size, shape, polydispersity) over colloidal nanoparticles is challenging because of the wide variety and interdependence of experimental input variables (e.g., reaction temperature, precursor concentration, reaction time, ligand concentrations, *etc.*) that can possibly affect these outcomes.^24^ Parsing the effects of experimental variables on specific synthetic outcomes, such as nanoparticle size, is traditionally done through one-variable-at-a-time (OVAT) exploration methods, which is inherently rooted in trial-and-error.^25^ OVAT methods are not only time- and labor-intensive and experimentally costly, but ultimately insufficient in quantitatively interpreting the complex, *n*-dimensional experimental variable space of a given synthesis, which is affected by *n*-number of variables and the possible interactions between one or more of those variables.^26,27^ Thus, data-driven techniques that enable rationally guided design space exploration of a given material synthesis are necessary to more rapidly gain efficient and accurate control over nanoparticle morphology and the resulting catalytic performance.^28^

One data-driven technique that can model such multivariate systems and uncover the effects of several experimental variables on a desired outcome is Bayesian optimization. This technique uses a surrogate model, built with existing literature and experimental data, to initially describe an objective function and its probability distribution within a defined design space. The objective function then guides an iterative optimization of a desired outcome by defining a set balance of exploiting the information provided by the data in the surrogate model and exploring the design space where data is lacking, until a global optimum is reached. Each time new experimental data is acquired, it is appended to the surrogate model to increase model accuracy. This type of iterative optimization is still in its relative infancy in the field of materials synthesis.^29−35^ That is, it has never been utilized for the complex optimization of a colloidal nanoparticle system with multiple responses as a function of multiple experimental variables while also incorporating a classifier to target a specific crystal phase.

Herein, we implement a multivariate Bayesian optimization in conjunction with a data-driven classifier to optimize a colloidal cobalt oxide nanoparticle synthesis, with the interdependent goals of decreasing the nanoparticle size to provide catalytically available surface-sites, and increasing the monodispersity in size and shape to enable investigation of structure–function relationships relevant for CO_2_ hydrogenation.^18,36^ The optimized small, phase-pure rock salt CoO nanoparticles with uniform cuboidal morphology were supported on SiO_2_ and evaluated for thermocatalytic CO_2_ hydrogenation in comparison to large, polydisperse CoO nanoparticles supported on SiO_2_ as well as a traditionally prepared catalyst. The controlled morphological characteristics had a beneficial effect on catalytically relevant properties and performance (i.e., both conversion and selectivity). The optimized CoO nanoparticles exhibited the highest activity of the catalysts tested (ca. 50% CO_2_ conversion), with high selectivity to methane (ca. 98%) across a range of catalyst pretreatment temperatures (300–450 °C). In contrast, the unoptimized CoO nanoparticles exhibited extremely low conversion (<1%), and a control CoO/SiO_2_ catalyst prepared by traditional incipient wetness impregnation demonstrated decreasing conversion and a shift from methane to CO selectivity with increasing pretreatment temperatures. These stark differences are attributed to the control of CoO particle size and shape, or lack thereof, and the resulting site densities for both reactants (i.e., CO_2_* and H*).

## Results and Discussion

A common method for the synthesis of colloidal transition metal oxide nanoparticles is the high-temperature reaction of a metal acetylacetonate (acac; C_5_H_7_O_2_^–^) precursor in the presence of long-chain aliphatic ligands, an alcohol or diol, and a high boiling solvent.^37^ The colloidal synthesis of cobalt oxide nanoparticles, adapted from previous literature methods,^38^ was performed through the reaction of Co(acac)_2_ in the presence of oleylamine (OAm; C_18_H_37_N), oleic acid (OA; C_18_H_34_O_2_), hexadecanol (C_16_H_34_O), and 1-octadecene. Increasing the surface-area-to-volume ratio (i.e., decreasing nanoparticle size) gives access to more catalytically available surface-sites,^39−42^ and increasing the monodispersity (i.e., decreasing the polydispersity and the shape variance) is important for producing a more uniform ensemble of nanoparticles, which can impact catalytic selectivity. The specific set of goals, or responses, that were chosen for the Bayesian optimization of this synthesis (i.e., the metrics by which the optimization will be assessed) are then: (1) minimization of nanoparticle size, (2) minimization of the particle distribution, and (3) minimization of shape variance. Minimizing these three separate responses for the target rock salt CoO phase is necessary to produce a well-defined nanoparticle catalyst. The input variables chosen as the most likely experimental variables to affect the outcome of these responses were based on an assessment of prior literature,^14,38,43−45^ and included reaction temperature (°C), reaction time (min), and the molar ratios of OAm:Co(acac)_2_, OA:Co(acac)_2_, and hexadecanol:Co(acac)_2_ (mol/mol).

After establishing the goals for each specific response and the input experimental variables, the first step in performing the Bayesian optimization was to construct the surrogate model, which uses experimental data that describes the defined parameter space. These data acted as a starting point for the initial training of the model to be optimized. The bounds of the parameter space must be determined because they define the edges of the experimental domain. The experimentally determined bounds of the surrogate model are given in Table 1. To collect the experimental data for the surrogate model, variables were then systematically explored within these bounds using orthogonal screening matrices. These statistical techniques are typically used in design of experiments (DoE), but can also be used to construct surrogate models, as they allow for the systematic sampling of a design space in a minimum number of experiments.^27^ The surrogate model consisted of 19 reactions that were performed from a full factorial DoE screening design for four variables (with reaction time being fixed at 60 min), 32 reactions from a Doehlert optimization matrix for five variables (now varying time), and 21 reactions that were data mined from the literature.^14,38,44−46^ In total, this resulted in 72 reactions, as detailed in the Supporting Information. The responses resulting from the product of each reaction were correspondingly characterized. The crystal phases of all the products were determined by powder X-ray diffraction (XRD). The nanoparticle size, polydispersity (σ/*d̅*), and shape variance were then quantified by transmission electron microscopy (TEM) using a previously published automated TEM image analysis pipeline,^47^ as described in the Supporting Information. The automated image analysis pipeline was used to assess the entire ensemble present in the inputted images (tens of thousands of particles, *vide infra*). This approach reduces selection bias, as any small or large particles present in the images are necessarily included in the size, polydispersity, and shape variance quantification.

**Table 1:** Surrogate Model Bounds

Experimental Variables	High Bound (+)	Low Bound (−)
Temperature (°C)	340	185
Reaction Time (min)	180	30
Oleylamine:Co(acac)_2_ (mol/mol)	200	0.5
Oleic acid:Co(acac)_2_ (mol/mol)	20	0
Hexadecanol:Co(acac)_2_ (mol/mol)	6	0

**Figure 1:**
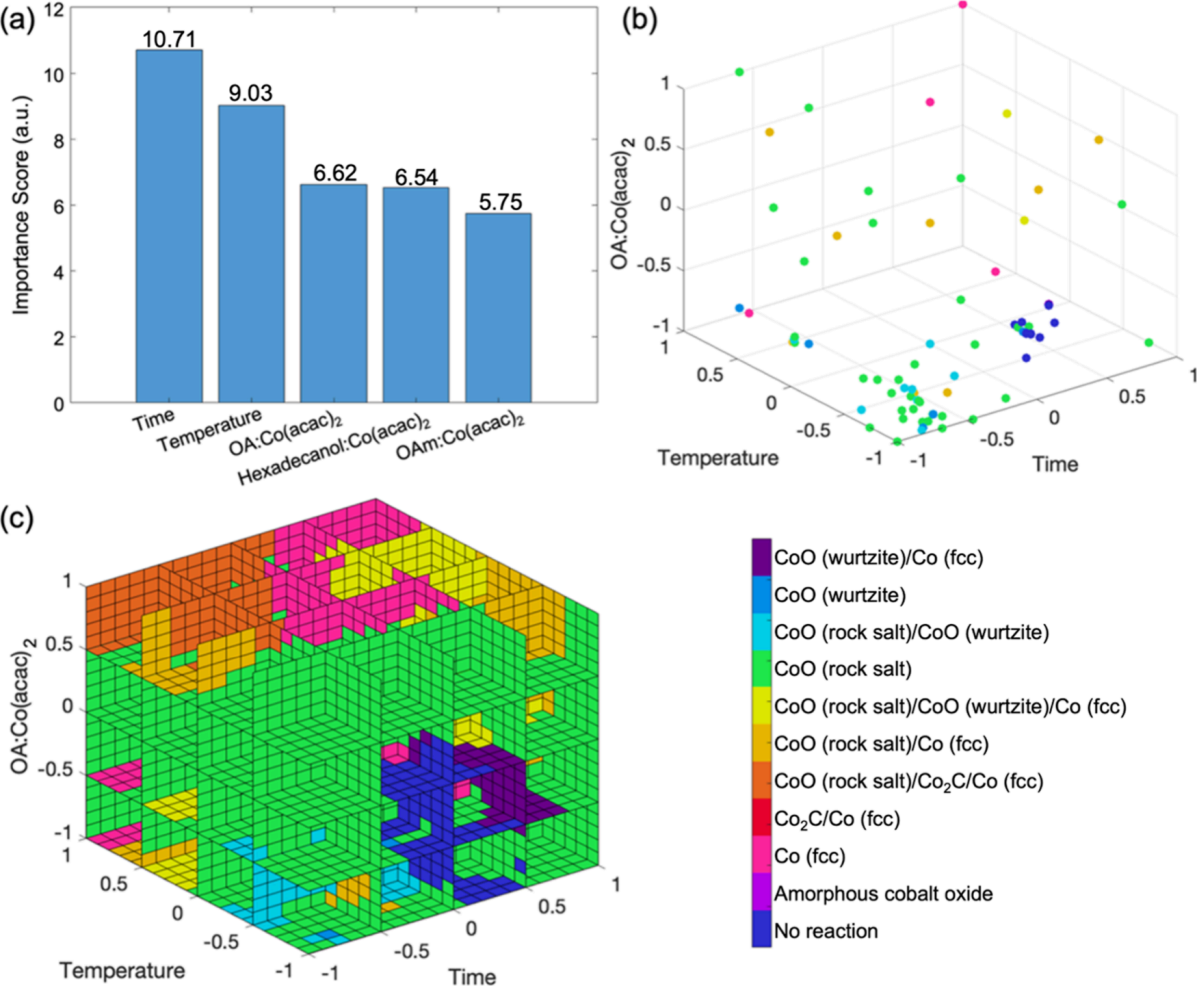
Phase map for the colloidal synthesis of rock salt CoO. (a) Importance scores of each experimental variable in determining crystal phase, with the reaction time, reaction temperature, and molar ratio of OA:Co(acac)_2_ having the greatest influence on phase determination. (b) Scatter plot of the reactions corresponding to these three most important experimental variables. (c) Predicted phase map after extrapolation corresponding to the three most important experimental variables. The resulting 10 phases or phase combinations are coded by color according to the key, with the target rock salt CoO phase given in green.

Upon compiling the experimental data for the surrogate model, 10 distinct crystal phases or phase combinations were observed in the products of the 72 reactions performed within the defined parameter space (Figure 1), as assessed by powder XRD. These included the target rock salt CoO phase, in addition to metastable wurtzite CoO, metallic fcc Co, cobalt carbide (Co_2_C), and phase combinations thereof. Some fraction of conditions resulted in amorphous material or no reaction, which is defined by a lack of isolable product. Combinations of crystalline and amorphous phases were not considered, as it is difficult to deconvolute amorphous inorganic phases from amorphous organic ligand content by powder XRD. Navigating such a complex phase space to optimize the morphology of only rock salt CoO nanoparticles is necessarily complex. Therefore, to enable synthetic control over the phase outcome to selectively produce rock salt CoO within the Bayesian optimization, the surrogate model data were used to train an ensemble classifier to predict crystal phase based on a given set of reaction conditions (see Supporting Information). Training a classifier was necessary due to the discrete categorical nature of phase as an outcome (i.e., there are a fixed integer number of possible phases).^48^ The effects of the chosen experimental variables on phase were subsequently analyzed, and importance scores were calculated for each variable based on their role in dictating crystal phase in the algorithm predictions. The variables with the greatest effects on crystal phase were reaction time, reaction temperature, and molar ratio of OA:Co(acac)_2_ (Figure 1a). Extrapolating the phase outcomes of the reactions plotted in Figure 1b using a nearest-neighbor likelihood algorithm yielded a prediction interpolant (i.e., a function that can be evaluated at query points) of 3-dimensional phase maps for each unique combination of the five variables (see Supporting Information). The phase map as a function of the three most important variables is illustrated in Figure 1c. To experimentally validate the phase map, a unique set of reaction conditions (not previously performed in the training set) lying within the binary phase space for rock salt CoO and metallic fcc Co was performed. With a OA:Co(acac)_2_ ratio of 10, a reaction time of 110 min, and a temperature of 263 °C (fixing the ratios of OAm:Co(acac)_2_ and hexadecanol:Co(acac)_2_ at 25 and 4, respectively), the reaction successfully produced a mixture of rock salt CoO and metallic fcc Co (Figure S15). The phase map was then used as the target reaction space, which defines the experimental variables that only lie within the target phase (i.e., rock salt CoO, coded as green within Figure 1c), as predicted by the classification algorithm.

Three Bayesian optimizations using an expected improvement algorithm and a modified rank-batch algorithm (detailed in the Supporting Information) were then performed to minimize nanoparticle size, size distribution of the ensemble (polydispersity), and shape variance within the target rock salt CoO phase space given in Figure 1c, as determined by the classifier (Scheme S1). The trained classifier was incorporated into the Bayesian optimizations by setting target phase as an initial condition for each iteration. For example, as the target was set to phase-pure rock salt CoO, each reaction predicted in the iteration would only continue in the optimization if the phase classifier predicted that set of reaction conditions to produce the correct phase. With each iteration, the model ran 30 test experiments and outputed a set of six unique reaction conditions, which were predicted to improve the accuracy of the model and therefore optimize the responses. This iterative cycle was performed a total of nine times, requiring 32 unique reactions to be carried out to acquire the conditions corresponding to a statistically significant, optimized rock salt CoO nanoparticle product.

**Figure 2:**
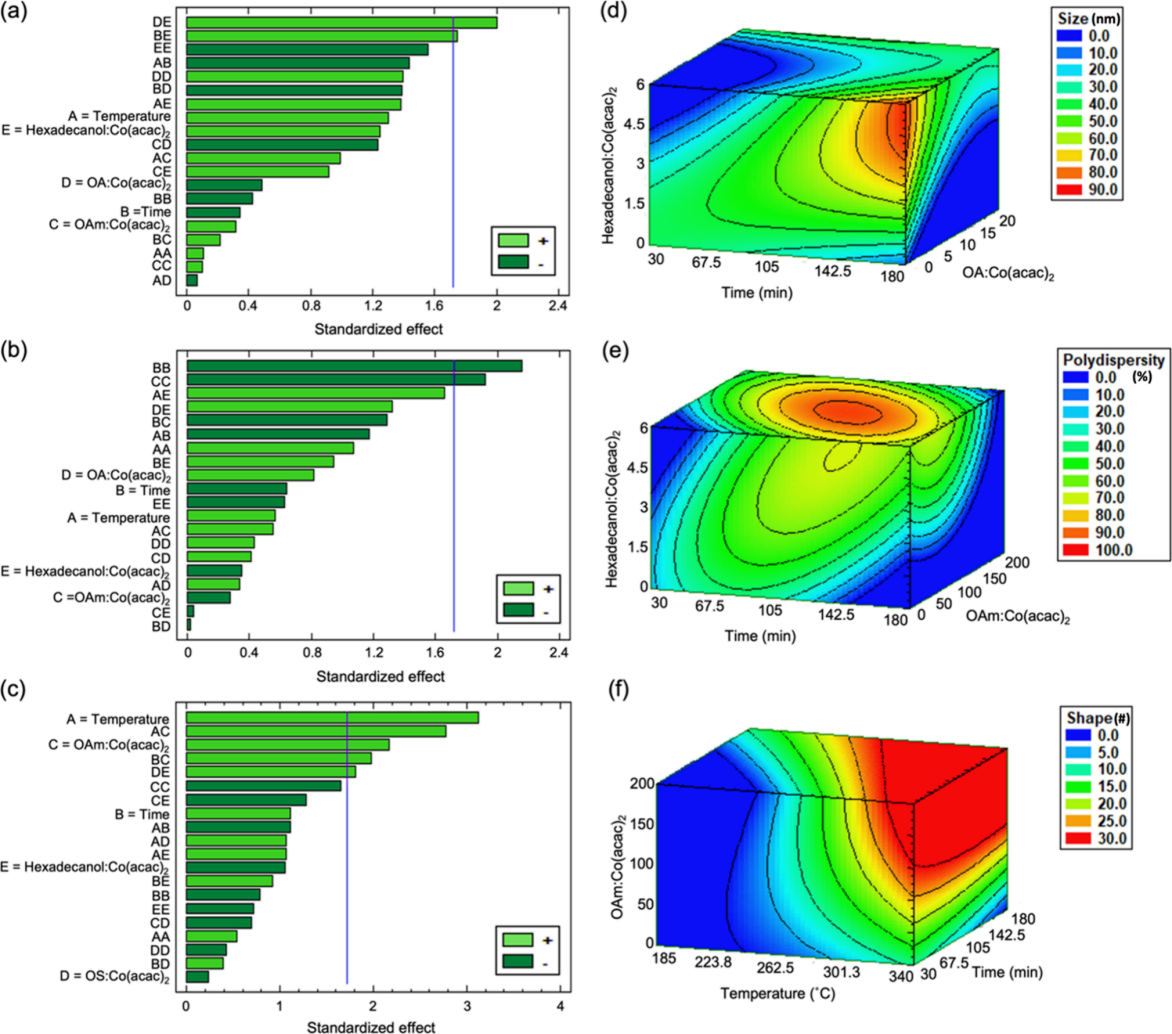
Pareto charts for statistical significance of the experimental variables that affect CoO nanoparticle (a) size, (b) polydispersity (σ/*d̅*), and (c) shape variance and their corresponding response surfaces for the three most important variables in (d), (e), and (f), respectively. The vertical blue lines in the Pareto charts represent the 90% confidence interval (α = 0.10).

The iterative process enabled elucidation of the role that each specific experimental variable plays in the optimization of the colloidal synthesis of rock salt CoO nanoparticles. The most important experimental variables for minimizing nanoparticle size were the second-order interactions between molar ratios of OA:Co(acac)_2_ and hexadecanol:Co(acac)_2_ and between reaction time and hexadecanol:Co(acac)_2_ ratio, as shown in the Pareto chart in Figure 2a. The length of the bar corresponding to each experimental variable, or combination of variables, in the Pareto chart is proportional to the value of a *t*-statistic calculated for the response. Any bars that exceed the vertical error line represent a statistically significant factor to the 90% confidence interval. The response surface for CoO nanoparticle size within the bounded parameter space for these three variables (molar ratios of OA:Co(acac)_2_ and hexadecanol:Co(acac)_2_ and reaction time) is given in Figure 2d, as determined by the model. The other two variables (reaction temperature and molar OAm:Co(acac)_2_ ratio) were fixed at the base level (i.e., 263 °C and 10, respectively). The most important experimental variables for the minimization of CoO nanoparticle polydispersity are the quadratic interactions at low levels of reaction time and molar OAm:Co(acac)_2_ ratio (Figure 2b), and the response surface for nanoparticle polydispersity is given in Figure 2e. The molar hexadecanol:Co(acac)_2_ ratio was chosen as the third variable in Figure 2e because of its significant interaction with both of the other two responses. The other two variables of reaction temperature and molar OA:Co(acac)_2_ ratio were fixed at the base level (i.e., 263 °C and 10, respectively). Finally, the most important variables for optimizing the CoO nanoparticle shape variance are the low levels of reaction temperature and molar OAm:Co(acac)_2_ ratio, the second-order interaction between those two variables, and the interaction between reaction time and molar OAm:Co(acac)_2_ ratio (Figure 2c), with the corresponding response surface given in Figure 2f. Here, the molar ratios of OA:Co(acac)_2_ and hexadecanol:Co(acac)_2_ were fixed at the base level (i.e., 10 and 3, respectively). As can be seen, the experimental variables that are the most significant in minimizing these three target goals are quite complex, given that there are many higher-order interaction effects. The elucidation of these variables would not be discernible using traditional methods and chemical intuition alone. This further illustrates the power of using data-driven methods to map a reaction parameter space efficiently and effectively for specific goals of a given synthetic optimization.

Nanoparticle size, polydispersity, and shape variance were then jointly optimized via a secondary Bayesian optimization, which used the models obtained from the iterative process. This optimization defined a multiobjective function to predict the set of conditions with the overall minimum score, using the most recent iteration from each response’s individual Bayesian optimizations as inputs. A desirability function was used to calculate the desirability of all three responses across the defined parameter space (see Supporting Information). Overall, the reaction time, reaction temperature, and molar ratio of OA:Co(acac)_2_ were determined to be the most important variables in the multiobjective optimization.

**Figure 3:**
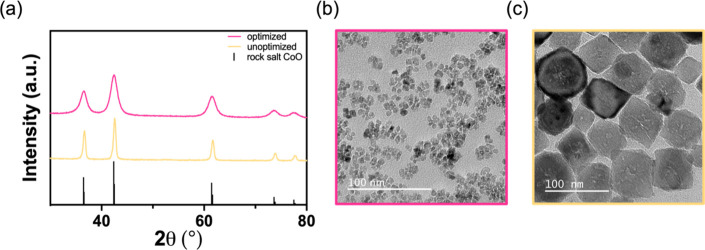
(a) Powder XRD patterns of optimized (pink) and unoptimized CoO (yellow) nanoparticles. The reference pattern for rock salt CoO (PDF #00–009–0402) is provided below. TEM images of (b) optimized and (c) unoptimized rock salt CoO nanoparticles. Both scale bars represent 100 nm.

The reaction conditions at the predicted multiobjective optimum were a temperature of 206 °C, a time of 54 min, a molar OA:Co(acac)_2_ ratio of 0.4, a molar OAm:Co(acac)_2_ ratio of 3.8, and a molar hexadecanol:Co(acac)_2_ ratio of 1.7 to produce a product with a predicted size of 9.8 ± 4.4 nm and a cuboidal particle morphology that belonged to four statistically significant shape groups, which is the lower bound of number of shape groups in order to avoid underfitting.^47^ The experimental validation of this prediction was performed in triplicate and the averaged results were in good agreement with the predicted responses, yielding rock salt CoO nanoparticles with a size of 6.6 ± 2.9 nm and four distinct shape groups, as assessed by automated TEM image analysis for the entire ensemble (*N* = 36,000), and 5.7 ± 0.9 nm when assessed by typical manual image analysis of *N* = 300 nanoparticles (Figure 3b).^49^ These experimentally validated rock salt CoO nanoparticles produced at the predicted optimum conditions can be compared to nanoparticles synthesized under unoptimized conditions (before the Bayesian optimization); these conditions yielded significantly larger rock salt CoO nanoparticles. The unoptimized reaction conditions used to produce large CoO nanoparticles were a temperature of 340 °C, a time of 60 min, a molar OA:Co(acac)_2_ ratio of 20, a molar OAm:Co(acac)_2_ ratio of 20, and a hexadecanol:Co(acac)_2_ ratio of 0.5. This reaction yielded nanoparticles with an average size of 65 ± 40 nm with nine distinct shape groups, as assessed by automated TEM image analysis for the entire ensemble (*N* = 36,000), and 68 ± 19 nm when measured by typical manual image analysis of *N* = 300 nanoparticles (Figure 3c).

The resulting nanoparticles were confirmed to be phase-pure rock salt CoO by powder XRD, with a lack of any significant amorphous background contribution (Figure 3a). Rietveld refinement of the resulting diffraction pattern using the cubic *Fm*3̅*m* space group returned a lattice constant of *a* = 4.2497(3) Å and a unit cell volume of *V* = 76.751 Å^3^ for the small, optimized CoO nanoparticles (Figure S17). This is in agreement with the previously published lattice parameter and unit cell volume for bulk rock salt CoO (*a* = 4.25 Å, *V* = 76.77 Å^3^, PDF #01–074–2391).^50^ Scherrer analysis of the XRD pattern returned an average grain size of ca. 5.5 nm for the optimized, small CoO nanoparticles, which is in reasonable agreement with the size obtained from TEM and suggests single crystalline particles. For the unoptimized, large CoO nanoparticles, Rietveld refinement of the XRD pattern also confirmed phase purity and returns a lattice parameter (*a* = 4.2565(9) Å) consistent with that of bulk rock salt CoO (Figure S18). Scherrer analysis of the XRD pattern gave an average grain size of ca. 17 nm, which is significantly smaller than the mean size obtained from TEM analysis, suggesting particle polycrystallinity (Figure 3c).^44^ Representative selected area electron diffraction (SAED) and high-resolution TEM (HR-TEM) images of both the optimized and unoptimized CoO nanoparticles are given in Figure S19 and further corroborate the formation of phase-pure, rock salt CoO. High-resolution TEM analysis of these CoO nanoparticles revealed lattice fringes, with the measured *d*-spacings of the small CoO nanoparticles *d* = 0.24 and 0.21 nm corresponding to the (111) and the (200) lattice planes, respectively.^51^ Similarly, the measured *d*-spacing of the large CoO nanoparticles *d* = 0.24 nm corresponds to the (111) lattice plane.

The optimized and unoptimized colloidal CoO nanoparticles were then dispersed on an amorphous SiO_2_ support (BET surface area of 190 m^2^/g) for characterization of catalytically relevant properties as a function of reductive pretreatment temperature and evaluation for thermocatalytic CO_2_ hydrogenation. The reduction temperature of CoO affects the relative ratios of reduced and oxidized cobalt species, which has been shown to impact the catalytic performance, including Fischer–Tropsch hydrocarbon synthesis.^17−21^ These materials are termed Opt-CoO/SiO_2_ and Unopt-CoO/SiO_2_ and had Co loadings of 7.4 and 7.5 wt %, respectively (9.4 and 9.5 wt % CoO), as measured by inductively coupled plasma-optical emission spectroscopy (ICP-OES). An additional catalyst for comparison was prepared by traditional incipient wetness impregnation of the same SiO_2_ support (termed IWI-CoO/SiO_2_) with a Co loading of 9.0 wt %, as measured by ICP-OES. Changes in the crystalline structure of the catalysts under reducing conditions were evaluated by in situ variable temperature powder XRD. For Opt-CoO/SiO_2_ in the range of 300–500 °C, a sharpening of the features associated with rock salt CoO (36.2, 42.2, 61.0° 2θ) was observed (Figure S20a). At 550–600 °C, a new diffraction peak at 43.9° 2θ became apparent that is attributed to the formation of fcc Co metal and is associated with a concomitant decrease in intensity of the features for rock salt CoO. For Unopt-CoO/SiO_2_, there were also no new phases identified in the XRD patterns until ca. 600 °C, at which point a peak associated with fcc Co metal (43.9° 2θ) is observed (Figure S20b). In contrast, the initial XRD pattern for IWI-CoO/SiO_2_ (Figure S20c) exhibited peaks at 31.2, 36.5, 59.5, and 65.4° 2θ attributed to spinel Co_3_O_4_. At 350 °C, the emergence of peaks at 42.2, and 61.0° 2θ indicated conversion to rock salt CoO and then to fcc Co at 500 °C, as indicated by a diffraction peak at 43.9° 2θ. Toward comparing catalytic performance of similar Co species, these XRD data demonstrate that all three materials contain rock salt CoO above 200 °C, followed by reduction of this phase to crystalline fcc Co metal at higher temperatues. The crystalline fcc Co metal was first observed in the XRD patterns of Opt-CoO/SiO_2_, Unopt-CoO/SiO_2_, and IWI-CoO/SiO_2_ at 550, 600, and 500 °C, respectively.

**Figure 4:**
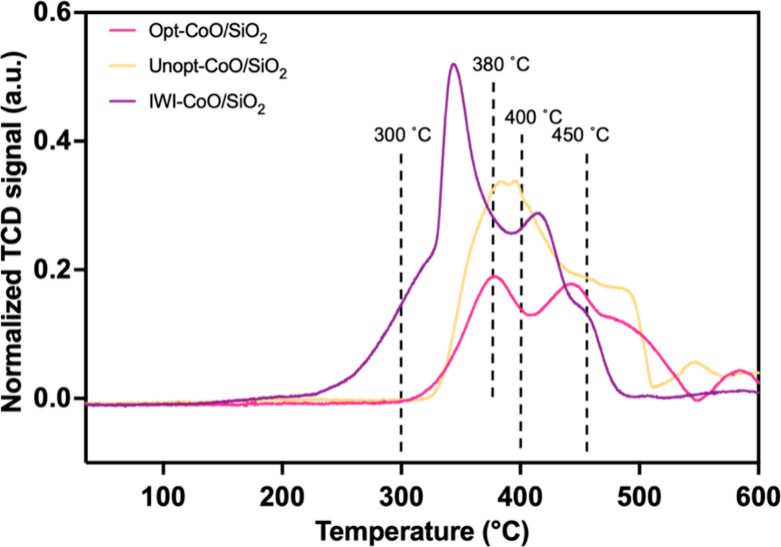
H_2_-TPR profile of supported CoO/SiO_2_ catalysts, with dashed lines indicating reduction events selected for CO_2_ hydrogenation catalytic activity testing.

To complement the understanding of crystal phases formed during in situ reduction observed by powder XRD, the reducibility of the three supported CoO/SiO_2_ catalysts was investigated using temperature-programmed reduction in flowing hydrogen (H_2_-TPR). As observed in Figure 4, multiple H_2_ consumption events occurred on all three catalysts over the range of 200–600 °C. The IWI-CoO/SiO_2_ catalyst exhibited a strongly rising edge of H_2_ consumption below 300 °C, which is attributed to the start of reduction of spinel Co_3_O_4_ to rock salt CoO, which peaks at 350 °C and is consistent with the transformation observed by XRD where crystalline CoO was observed at 350 °C. The H_2_ consumption event centered at 422 °C and extending to 500 °C can be assigned to the reduction of CoO to metallic Co, which is consistent with observed crystalline Co metal in the XRD pattern at 500 °C.^52^ In contrast, no reduction occurred over the Opt-CoO/SiO_2_ and Unopt-CoO/SiO_2_ catalysts at temperatures below 300 °C (i.e., the initial structures were rock salt CoO). For the Opt-CoO/SiO_2_ catalyst, two separate events were observed at 380 and 440 °C, followed by continued H_2_ consumption to 550 °C. The event at 380 °C may correspond to ligand decomposition, since it is not associated with reduction events leading to crystal phase changes in the XRD patterns. The defined reduction event at 440 °C is attributed to the formation of metallic Co clusters followed by bulk reduction at higher temperatures, consistent with the observation of fcc Co in the XRD pattern at 550 °C. For the Unopt-CoO/SiO_2_, the major H_2_ consumption event was observed between 325 and 450 °C, with continued H_2_ consumption to 500 °C. Similar to the Opt-CoO/SiO_2_, the events between 380 and 450 °C are attributed to ligand decomposition without any crystal phase change, and the broad reduction event at higher temperatures is associated with the reduction of CoO to Co metal, where crystalline fcc Co was observed at 600 °C by XRD. The breadth of the reduction peak associated with CoO reduction to Co metal for the Unopt-CoO/SiO_2_, may be related to the inhomogeneity of the particles, as particle size has been shown to impact reduction temperature.^53^ It is worth noting that these H_2_-TPR data do not inform ligand removal effectiveness, which may be difficult to investigate due to differences in CoO particle size and ligand coverage on both the colloidal nanoparticles and the support, and therefore, residual carbonaceous species may be left behind after reduction (*vide infra*). From these data, specific reduction temperatures of 300, 380, 400, and 450 °C were selected to study the effect of reduction temperature on catalyst performance in the CO_2_ hydrogenation reaction. The extremes of these pretreatment temperatures (i.e., 300 and 450 °C) were chosen to assess active site densities for both CO_2_ and H_2_.

Active site densities of the three CoO/SiO_2_ catalysts were measured using H_2_ and CO_2_ chemisorption after reductive pretreatment at 300 or 450 °C. All three catalysts exhibited strong CO_2_ adsorption. In contrast, although the catalysts did activate H_2_ at the analysis temperature of 50 °C, it was weakly bound, leading to low or negligible strong H* site density values. To compare the site densities across this series of materials, the site densities for strong CO_2_ adsorption (μmol-CO_2_*/g_cat_) and total H_2_ adsorption (μmol-H*/g_cat_) are reported in Table 2, where the total H_2_ value includes contributions from both weak and strong binding H*. The Unopt-CoO/SiO_2_ catalyst exhibited low site densities for both CO_2_* and H*. Negligible H_2_ adsorption was observed at all reduction conditions, and the CO_2_* site density was ca. 15–20% of that demonstrated by the IWI-CoO/SiO_2_ and Opt-CoO/SiO_2_ catalysts. The Opt-CoO/SiO_2_ catalyst exhibited similarly high CO_2_* site density values after reduction at 300 and 450 °C (77.5 and 80.0 μmol-CO_2_*/g_cat_, respectively). A moderate increase in CO_2_* site density was observed for the IWI-CoO/SiO_2_ catalyst with increasing pretreatment reduction temperature (from 65.9 to 92.5 μmol-CO_2_*/g_cat_ at 300 and 450 °C, respectively). Nevertheless, the CO_2_* site density was in a comparable range of 65–90 μmol-CO_2_*/g_cat_ for the IWI-CoO/SiO_2_ and Opt-CoO/SiO_2_ catalysts across the investigated pretreatment reduction conditions. In contrast, the H* site densities were markedly different for these two catalysts. The IWI-CoO/SiO_2_ catalyst exhibited a low H* site density after a 300 °C reduction (4.8 μmol-H*/g_cat_), that then decreased to zero after the 450 °C reduction. This result suggests possible sintering of metallic Co species in the IWI-CoO/SiO_2_ catalyst leading to low metal surface area and negligible H_2_ activation.^54^ The Opt-CoO/SiO_2_ catalyst exhibited an H* site density of 15.5 μmol-H*/g_cat_, which is 3× greater than that for the IWI-CoO/SiO_2_ catalyst. A slight increase to 16.8 μmol-H*/g_cat_ was observed after the higher temperature reduction (450 °C), in stark contrast to the negligible H_2_ activation exhibited by the other catalysts after this higher temperature reduction. This behavior indicates a more stable metallic Co or CoO_1–*x*_ species that does not sinter after the higher temperature reduction for the Opt-CoO/SiO_2_ catalyst.

**Table 2:** Chemisorption Data for Strong CO_2_* and Total H* Site Densities on CoO/SiO_2_ Catalysts Measured at 50 °C After H_2_ Reduction at 300 or 450 °C

		Site Density (μmol-CO_2_*/g_cat_ or μmol-H*/g_cat_)		
Reduction Temp. (°C)	Probe Molecule	Opt-CoO/SiO_2_	Unopt-CoO/SiO_2_	IWI-CoO/SiO_2_
300	CO_2_	77.5	13.2	65.9
	H_2_	15.5	0	4.8
450	CO_2_	80.0	4.4	92.5
	H_2_	16.8	0	0

X-ray photoelectron spectroscopy (XPS) was performed on the as-synthesized Opt-CoO/SiO_2_ and Unopt-CoO/SiO_2_, and then following reduction at 450 °C to confirm the presence of metallic Co species. The reduced catalysts were transferred to the vacuum environment of the XPS system without exposure to air. Spectra were energy-referenced by centering the silicon 2*p* envelope on 103.5 eV.^55^ The cobalt 2*p*_3/2_ spectra of the as-synthesized catalysts exhibit two main features centered on 781 and 786 eV (Figure S21). In contrast, the Opt-CoO/SiO_2_ and Unopt-CoO/SiO_2_ catalysts that were reduced at 450 °C exhibited additional components at lower binding energy (776–778 eV). Although quantitative interpretation of first row transition metal 2*p* spectra is far from straightforward, it is clear that the lowest binding energy component in the spectra of the reduced catalysts is only explainable on the basis of there being some amount of metallic cobalt in these samples.^56^ The fact that these metallic species appear in XPS at 450 °C and that XRD shows crystalline Co metal appearing at ∼550 °C implies that metallic cobalt initially forms in very small clusters and/or an amorphous state.

**Figure 5:**
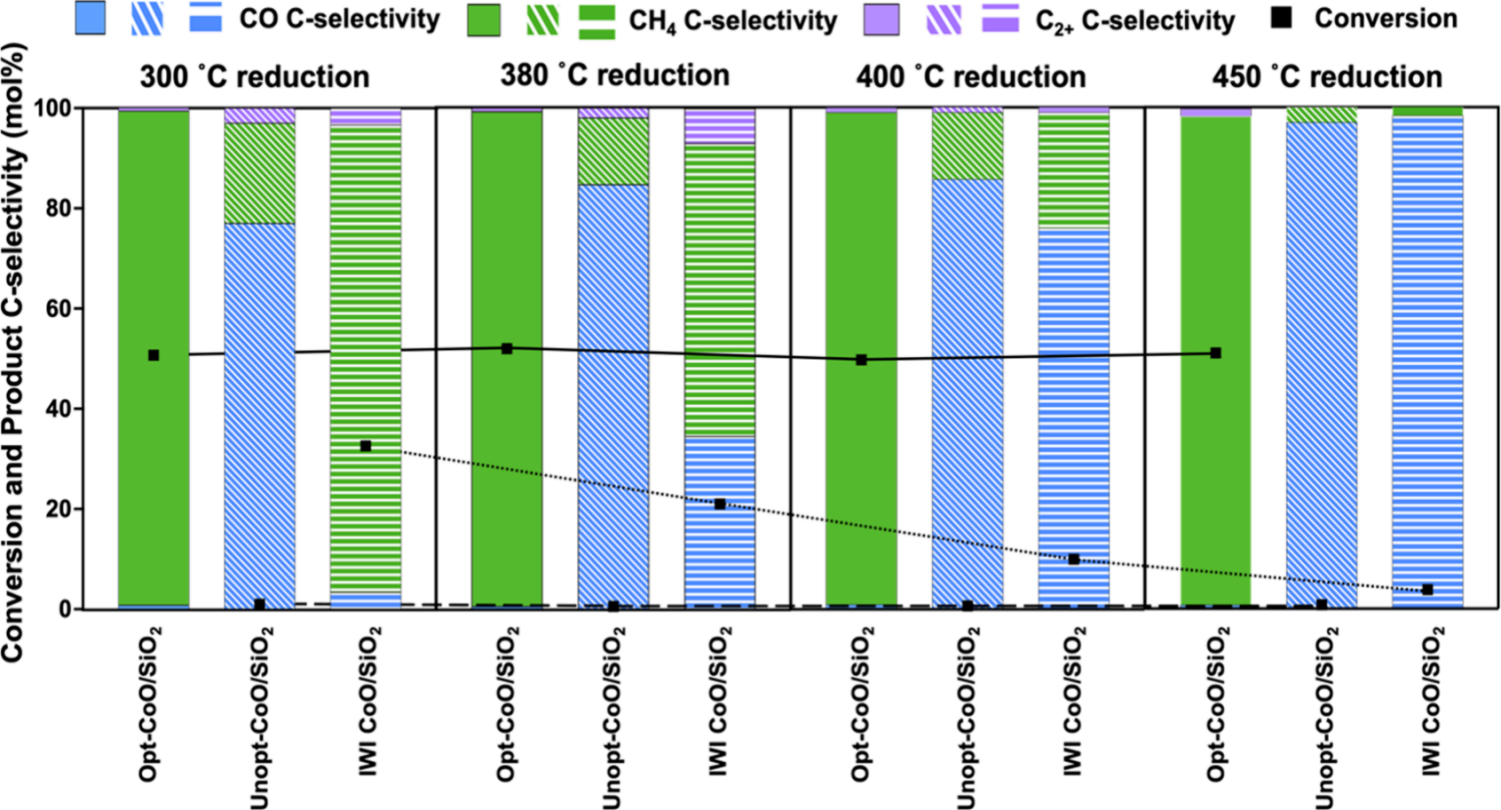
Catalytic performance of CoO/SiO_2_ catalysts in the CO_2_ hydrogenation reaction after reductive pretreatments between 300 and 450 °C. Reaction conditions were 300 °C, 3 MPa, WHSV = 1 g CO_2_·g·cat^–1^·h^–1^, and H_2_:CO_2_ molar ratio of 3. Square symbols are conversion; blue bars are CO C-selectivity; green bars are CH_4_ C-selectivity; purple bars are C_2+_ hydrocarbon C-selectivity.

Catalytic activity of the CoO/SiO_2_ catalysts was evaluated in the CO_2_ hydrogenation reaction with an H_2_:CO_2_ mol/mol ratio of 3. The catalysts were pretreated at the four temperatures identified by H_2_-TPD in flowing H_2_, and the reaction was performed at 300 °C and 3 MPa for at least 7 h after each pretreatment condition. The total time to complete the series of experiments over each catalyst bed was approximately 70 h, and the time-on-stream data for each catalyst is provided in Figures S22–24. Catalytic performance metrics (i.e., conversion and C-selectivity) are reported as the average from the last 3 h of reaction time (Figure 5). The Unopt-CoO/SiO_2_ catalyst exhibited extremely low CO_2_ hydrogenation activity, with the overall conversion <1% at all conditions. As the reduction temperature increased from 300 to 450 °C, selectivity to CO increased from 76.9% to 96.1%, while CH_4_ selectivity decreased concomitantly from 19.4% to 3.4%. The IWI-CoO/SiO_2_ catalyst exhibited hydrogenation activity after the low reduction temperature (300 °C) with 32.6% conversion and 93.3% selectivity to CH_4_. As the reduction temperature increased to 450 °C, however, conversion decreased significantly to 3.8% and the dominant product was CO with selectivity of 98.3%, while CH_4_ selectivity decreased to 1.6%. Interestingly, the Opt-CoO/SiO_2_ catalyst maintained high hydrogenation activity over the entire range of reduction temperatures investigated here. After the 300 °C reduction, the CO_2_ conversion was 50.7% and the major product was CH_4_ with a selectivity of 98.6%. As the reduction temperature increased from 300 to 450 °C, the conversion was maintained in the range of 49.7–52.0% and CH_4_ was always the dominant product with nearly constant selectivity in the range of 97.5–98.6%. Low selectivity to C_2+_ hydrocarbons was observed via Fischer–Tropsch chemistry under these conditions. For the nanoparticle catalysts, these products were predominantly ethane, ethylene, propane, and propylene, with additional products of butenes and pentenes observed at low selectivity for the IWI-CoO/SiO_2_ catalyst.

**Figure 6:**
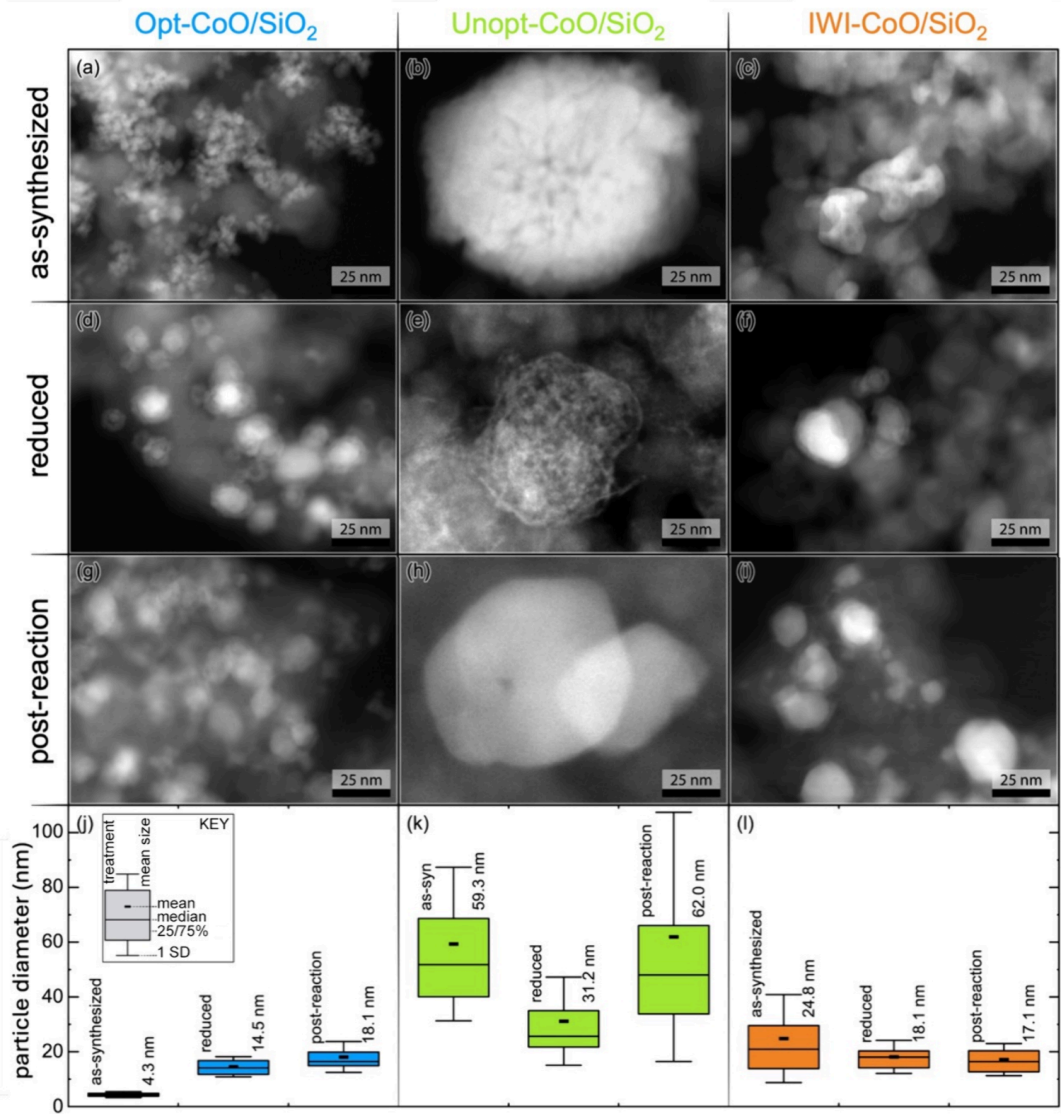
STEM-HAADF images of Opt-CoO/SiO_2_, Unopt-CoO/SiO_2_, and IWI-CoO/SiO_2_ catalysts in the (a–c) as-synthesized forms, (d–f) following reduction at 450 °C, and (g–i) after CO_2_ hydrogenation with a 450 °C reductive pretreatment, as well as (j–l) associated particle diameter distributions. All scale bars represent 25 nm.

To provide insight into particle size evolution that could impact site densities and catalytic performance, scanning transmission electron microscopy (STEM) with a high angle annular dark field (HAADF) detector and energy dispersive spectroscopy (EDS) was performed on the catalysts in the as-synthesized forms, following reduction at 450 °C, and after CO_2_ hydrogenation with a 450 °C reductive pretreatment. Figure 6 provides representative HAADF-STEM images as well as associated particle size distributions. HAADF-STEM is highly sensitive to atomic number, so brighter regions in the STEM micrographs correspond to cobalt localization, while greyer regions indicate silica and carbonaceous species. The as-synthesized Opt-CoO/SiO_2_ and Unopt-CoO/SiO_2_ catalysts had average CoO particle diameters of 4.3 ± 1.1 nm and 59.3 ± 27.9 nm, respectively, which agreed well with those measured from the TEM images of the unsupported colloidal nanoparticles (Figure 3). Following reduction at 450 °C, the average particle diameter in the Opt-CoO/SiO_2_ increased to 14.5 ± 3.7 nm, likely due to some sintering of nearby particles, while the average particle diameter in Unopt-CoO/SiO_2_ decreased to 31.2 ± 16.0 nm, with significant overlap in the size distributions before and after reduction for the unoptimized catalyst. Notably, the appearance of the nanoparticles in the reduced Unopt-CoO/SiO_2_ catalyst differed significantly from those in the as-synthesized catalyst. The particles in the reduced Unopt-CoO/SiO_2_ catalyst developed a lacey structure following reduction (Figure 6e) that was consistent throughout the entire supported catalyst. The regions of higher contrast in the lacey structured particles may contain higher cobalt density resulting from reduction of the polycrystalline Unopt-CoO nanoparticles. In contrast to the particles in the reduced Opt-CoO/SiO_2_ catalyst, those in the reduced Unopt-CoO/SiO_2_ catalyst were also found by HAADF-STEM-EDS analysis imaging to have a lower contrast shell that did not contain cobalt (Figure S25). Residual organic species from synthesis of the polycrystalline Unopt-CoO nanoparticles may not have been completely removed during reduction and instead formed a carbon-containing shell. In addition to the larger size of the cobalt-containing particles in Unopt-CoO/SiO_2_, carbon contamination on the surface of the particles could contribute to the low measured strong CO_2_* and total H* site densities and resulting limited CO_2_ conversion at all pretreatment temperatures for the Unopt-CoO/SiO_2_ catalyst. The particles in the IWI-CoO/SiO_2_ catalyst underwent a decrease in the apparent average particle diameter observed via STEM, from 24.8 ± 16.0 nm to 18.1 ± 6.0 nm (Figure 6c,f) upon reduction at 450 °C. Finally, HAADF-STEM analysis of the catalysts after CO_2_ hydrogenation with a 450 °C reductive pretreatment (Figure 6g-i) did not show a dramatic increase in average particle diameter for any catalyst, although the particles in the Unopt-CoO/SiO_2_ no longer exhibited the lacey structure observed in the reduced material, indicating sintering of the polycrystalline material during catalysis. Analysis of the postreaction catalysts by scanning electron microscopy (SEM) with EDS was also performed to identify any populations of larger cobalt-containing particles that may not have been observed by higher resolution STEM analysis. The SEM images and EDS elemental maps provided in Figure S26 indicate that postreaction Opt-CoO/SiO_2_ catalyst has well-distributed cobalt species with no discernible aggregates, while the Unopt-CoO/SiO_2_ shows some evidence of larger submicron cobalt-containing aggregates. In contrast, the IWI-CoO/SiO_2_ includes relatively large cobalt aggregates in the 1–5 μm range that likely contribute to the lower catalytic activity of the catalyst.

The change in activity and selectivity after increasing reduction temperature for these three catalysts can be rationalized by considering the CoO features observed by HAADF-STEM-EDS and chemisorption results reported above. The low activity observed from the Unopt-CoO/SiO_2_ catalyst is consistent with the low CO_2_* site density and lack of H* sites after either pretreatment condition, and these could be attributed to the large CoO particle size (and relatively lower exposed surface area) as well as the presence of a carbon shell blocking surface sites. For the IWI-CoO/SiO_2_ catalyst, the observed drop in conversion and selectivity shift from CH_4_ to CO after higher reduction temperatures is consistent with the observed decrease in surface H* coverage with increasing temperature (i.e., giving an increasing ratio for CO_2_*/H*). The CO_2_* site density values suggest that surface CO_x_* species would remain available after high temperature reduction, but there would be less H* available, leading to decreased hydrogenation activity, lower selectivity to CH_4_, and higher selectivity to CO. This effect of surface CO_2_*/H_2_* ratio on CO and CH_4_ selectivity has been reported for supported Co species, and for supported carbide catalysts by our group.^57−60^ In contrast, the Opt-CoO/SiO_2_ catalyst exhibited consistently high CH_4_ selectivity (i.e., consistent hydrogenation activity) across the pretreatment reduction temperature range studied here, and this is attributed to particle stability and the consistent H* surface coverage even after the higher temperature reduction (i.e., a constant ratio for CO_2_*/H*). Through the synthetic optimization of the colloidal nanoparticle morphology, a more stable Opt-CoO/SiO_2_ catalyst was obtained that does not sinter or become occluded by carbon after the higher temperature reduction.

## Conclusions

We report the first example of a multivariate Bayesian optimization coupled with a trained classifier to successfully optimize a colloidal nanoparticle synthesis while simultaneously targeting a specific crystal phase. Interfacing these two data-driven techniques with the goal of targeting phase-pure rock salt CoO while minimizing nanoparticle size, polydispersity, and shape variance resulted in a prescriptive set of reaction conditions that experimentally yielded small, monodisperse, and phase-pure rock salt CoO nanoparticles. The Bayesian optimization progressed through nine iterations, in which a multivariate joint optimization was performed to find the global optimum of all three target responses in only 32 reactions. The resulting optimized material had a 10-fold decrease in size, a 50% decrease in particle polydispersity, and a 75% decrease in shape variance compared to an unoptimized sample before the Bayesian optimization. The experimental validation of these reaction conditions was performed in triplicate and were in good agreement with the predicted responses, yielding nanoparticles with an average size of 6.6 ± 2.9 nm with four distinct shape groups, as assessed by automated TEM image analysis for the entire ensemble. This optimization was performed in conjunction with a classification algorithm that was successful in targeting the rock salt phase of CoO out of 10 unique phases or phase combinations that were observed during the initial screening design.

These results enable a route to systematically improve specific target goals by efficiently mapping a multivariate parameter space of a target material. For example, within the scope of nanoparticle catalysts, it is known that size and polydispersity can have a dramatic effect on catalytic activity and selectivity. The data reported here for CO_2_ hydrogenation reinforces this concept, where CoO particle size is linked to active site density, activity, and selectivity to CO or CH_4_. For the range of experimental variables that were investigated for the CoO nanoparticles herein, the optimization allowed us to view the entire parameter space of this material across the ranges of the five variables tested. This revealed that the reaction time, reaction temperature, and molar OA:Co(acac)_2_ ratio were the most important variables in the overall optimization. Such maps have the potential to enable more efficient structure/morphology-function relationships to be elucidated for a vast array of nanoparticle catalysts.

## Experimental Procedures

### Colloidal CoO Nanoparticle Synthesis

Oleylamine (70% technical grade), oleic acid (90% technical grade), and 1-octadecene (ODE, 90%) were purchased from Sigma-Aldrich and dried under vacuum at 120 °C for 5 h before use. Cobalt(II) acetylacetonate (Co(acac)_2_, 99%) and 1-hexadecanol (99%) were purchased from Sigma-Aldrich and were used as received. In a typical experiment, appropriate amounts of Co(acac)_2_ and hexadecanol were weighed out and added to a three-neck round-bottom flask, equipped with a reflux condenser, two septa, and a gas inlet adaptor. The flask was attached to a Schlenk line and evacuated and filled with nitrogen for three cycles. Upon the last refill with nitrogen, appropriate volumes of oleylamine, oleic acid, and ODE were injected into the flask and then the flask was heated rapidly to the set temperature in a temperature-controlled sand bath. Once the set temperature was reached, the reaction was held for a specific amount of time before removing the flask from the sand bath to quench by cooling naturally to ambient temperature in air. After cooling, the nanoparticle suspension was transferred to a 50 mL centrifuge tube with ca. 1 mL of hexanes added to the flask to assist with the transfer. Ethanol was added to the centrifuge tube to precipitate the nanoparticles followed by centrifugation (6,000 rpm, 10 min). The nanoparticle pellet was redispersed in a minimal amount of hexanes (ca. 1 mL) and precipitated again with ethanol. This purification cycle was repeated once more for a total of three washes. Upon the final wash, the nanoparticle pellet was redispersed in hexanes or dried under flowing nitrogen for further characterization.

### CoO Supported on SiO_2_

The silica support (Sipernat-22, *S*_BET_ 190 m^2^/g) was provided by Evonik and calcined at 500 °C for 5 h prior to use. Upon the final wash in the procedure above, 400 mg (ligand corrected mass via TGA) of the isolated CoO nanoparticles were redispersed in ca. 10 mL of CHCl_3_ and then added dropwise to a rapidly stirring (1,000 rpm) suspension of 4.0 g of SiO_2_ in *ca*. 40 mL CHCl_3_. The solution was bath sonicated for 5 min and then left to stir overnight at room temperature. The supported nanoparticles were collected via centrifugation (6,000 rpm, 5 min) and dried under flowing nitrogen.

### Synthesis of CoO/SiO_2_ by Incipient Wetness Impregnation (IWI)

The IWI-CoO/SiO_2_ catalyst was prepared using traditional IWI synthesis methods targeting a 10% loading of Co on the amorphous silica support (Sipernat-22). Briefly, an aqueous solution containing Co(NO_3_)_2_•6H_2_O in the appropriate concentration was added dropwise to the SiO_2_ powder. The impregnated material was dried in air at 110 °C for ca. 12 h. The dried material was then calcined in air by heating to 400 °C at 10 °C min^–1^ and maintaining the final temperature for 3 h.

### Inductively Coupled Plasma-Optical Emission Spectroscopy (ICP-OES)

The Co loading of the SiO_2_ supported CoO nanoparticles was determined by ICP-OES performed by Galbraith Laboratories (Knoxville, TN). The Co loading of the calcined IWI-Co/SiO_2_ catalyst was determined by ICP-OES at NREL. Around 250 mg the solid material was mineralized in a Teflon tube with 2 mL of concentrated HNO_3_, 1 mL of concentrated fluoroboric acid, and 5 mL of concentrated hydrochloric acid using a microwave digestion system (UltraWAVE 2, Milestone) at 1500 W following the UW-GE-4 method provided by Milestone. The sample was then diluted to 50 mL with distilled water with 0.25 mL of 1000 ppm yttrium in 2% HNO_3_ (Accustandard; New Haven, CT) added to serve as an internal standard for analysis by ICP-OES (ICP-OES 5100; Agilent Technologies Inc.).

### Temperature-Programmed Reduction (TPR)

Reducibility of the supported catalysts was studied by temperature-programmed reduction with H_2_ (H_2_-TPR) using an Altamira AMI-Lite instrument. Samples of ca. 50 mg of silica-supported material was loaded into a quartz u-tube reactor and dehydrated at 100 °C in Ar flow at 25 ccm for 4 h. After cooling to 25 °C, the sample was heated at a rate of 5 °C min^–1^ from 25 to 700 °C in 4% H_2_/Ar with a flow of 25 ccm, followed by a 1 h hold at 700 °C. The H_2_ concentration was monitored by a thermal conductivity detector (TCD) and H_2_ consumption was quantified by calibrating with 10 pulses of Ar from a 0.5 mL sample loop in a flow of 4% H_2_/Ar at 25 ccm. The signal was normalized to the mass of catalyst.

### Chemisorption

Volumetric chemisorption analyses were performed using a Quantachrome Instruments Autosorb-1C gas sorption instrument. A catalyst sample (120–200 mg of CoO/SiO_2_) was diluted with 1 g of quartz chips and loaded into a quartz u-tube reactor. The sample was reduced in pure H_2_ at 300 °C with a heating rate of 5 °C min^–1^, held for 2 h, and subsequently evacuated for 8 h at this temperature. After cooling to 50 °C, combined and weak H_2_ chemisorption isotherms were measured in the range of 100–600 Torr. The same catalyst sample was then reduced in pure H_2_ at 450 °C with the same ramp rate, hold time, and evacuation time, and another H_2_ chemisorption analysis was performed. The same pretreatment and experimental procedures were performed to measure CO_2_ chemisorption at 50 °C using a new catalyst load. The site density for strong CO_2_* (units of μmol_CO2*_/g_cat_) was determined from the difference of the combined and weak isotherms extrapolated to zero pressure. The site density for total H* (units of μmol_H*_/g_cat_) was determined from extrapolation of the combined isotherm to zero pressure.

### Catalytic Evaluation

Performance of the CoO/SiO_2_ catalysts in the CO_2_ hydrogenation reaction was evaluated in a tubular fixed-bed reactor (7.9 mm I.D.) at 300 °C and 3 MPa. The catalyst bed (comprising approximately 1 g of catalyst) was positioned within an isothermal region of the reactor, and the remaining reactor volume was packed with two particle sizes of crushed quartz. Approximately 2 mL of fine-crushed quartz (150–250 μm) was packed above and below the catalyst bed, and the remaining reactor volume was packed with course-crushed quartz (300–425 μm). The temperature of the isothermal zone was measured with a 4-point thermocouple inserted into the catalyst bed. The catalyst was pretreated under 95% H_2_/5% Ar flow (100 sccm) at atmospheric pressure and 300 °C for 4 h prior to reaction. After the reactor temperature was stabilized at 300 °C, reactant gas flow rates (including CO_2_ and 95% H_2_/Ar) were adjusted to achieve a weight hourly space velocity (WHSV) of 1.0 g_CO2_•g_cat_^–1^•h^–1^ (referred to as h^–1^) with a H_2_/CO_2_ molar ratio of 3, and the reactor was pressurized to 3 MPa to start reaction. At the completion of the reaction period, CO_2_ flow was stopped, and the reactor was depressurized and purged in 95% H_2_/Ar flow (100 sccm) for 8 h to remove residual CO_2_. Then, the reactor temperature was adjusted to the next reduction temperature. The same procedure was utilized to evaluate catalytic activity at four reduction temperatures (300, 380, 400, and 450 °C) on the same catalyst bed. After each pretreatment condition, the CO_2_ hydrogenation reaction was held for at least 7 h to achieve stable conversion for a period of 3 h. The cumulative time to complete the series of catalytic experiments after each reduction temperature was approximately 70 h.

Product analysis was performed online using an Agilent Technologies 7890B gas chromatograph equipped with flame ionization detectors (FIDs) to analyze oxygenates and hydrocarbons, and TCDs to analyze permanent gases. Reactor inlet and outlet gases were sampled through heated (200 °C) lines to prevent condensation prior to analysis. The concentration of each compound was quantified by correlating its peak area with the response factor obtained from traceable gravimetric calibration standards. Sampling of the inlet stream was also performed when the reactant flow was started or changed to measure concentration of the feed stream. Ar in the inlet stream was used as an internal standard to quantify molar flow rate of all other components in the gas stream. Conversion was calculated as ∑ (molar flow rate of C in all products)/(molar flow rate of inlet CO_2_) * 100 (%), while C-selectivity of product *i* was calculated as (molar flow rate of C in product *i*)/∑ (molar flow rate of C in all products) * 100 (%).

### Powder X-ray Diffraction (XRD)

Powder XRD patterns were collected on a Rigaku Ultima IV diffractometer with a Cu *Kα* X-ray source (λ = 1.5406 Å), operating at 40 mA and 44 kV. Rietveld refinements were performed using GSAS-II.^61^ ICSD structural files of rock salt CoO were used to fix the experimental data. The profile parameters *U* and *X* were refined. A total of 10 fixed background coefficients were used with a Chebyschev polynomial function to fit the background contribution. The lattice parameters, surface displacement, surface roughness, and crystalline size were also refined. The *R*_wp_ percent and χ^2^ indicators were used to define the quality of the refined structural models.

### In Situ Powder X-ray Diffraction (XRD)

In situ X-ray powder diffraction data were collected using a Rigaku Ultima IV diffractometer with a Cu *Kα* source (40 kV, 44 mA) fitted with a Reactor X high temperature reaction cell. Diffraction patterns were compared to powder diffraction files from the International Centre for Diffraction Data (rock salt CoO: 00–009–0402, fcc Co metal: 00–015–0806, spinel Co_3_O_4_: 01–080–1532). Line positions were normalized to an external Si reference (NIST Standard Reference Material 640A). A portion of CoO/SiO_2_ was pressed into the quartz sample holder and placed in reaction chamber. Diffraction patterns were collected in the 2θ range of 20–85° at a scan rate of 4° min^–1^. For data collection under a reducing environment the reaction chamber was first purged for at least 15 min with inert gas at 40 sccm. The gas flow was then changed to the 5% H_2_ process gas (H_2_, 2 sccm; He, 38 sccm). A baseline pattern was collected at 35 °C then the sample was heated to 600 °C at 10 °C min^–1^ before cooling to ambient temperature. Patterns were collected every 50 °C after (and including) 100 °C, waiting 10 min after data collection before resuming the temperature ramp (ca. 30 min residence at each temperature point).

### Transmission Electron Microscopy (TEM)

TEM images were acquired with a JEOL JEM2100F (JEOL Ltd.) microscope operating at 200 kV. Each sample was prepared by drop-casting a hexanes suspension of the nanoparticles onto 400 mesh Cu grids coated with a lacey carbon film (Ted Pella, Inc.) and dried overnight under vacuum at room temperature. Scanning transmission electron microscopy (STEM) with a high angle annular dark field (HAADF) detector was performed with a Spectra200 S/TEM (ThermoFisher Sciences) equipped with an X-CFEG source and operating at an acceleration voltage of 200 kV. STEM is highly sensitive to *Z*-number, so brighter regions in the STEM micrographs correspond to cobalt localization, while greyer regions indicate silicon and carbon species. Samples were prepared via suspension in hexanes followed by ultrasonic bath treatment for 5 min to improve particle dispersion. Immediately prior to analysis, the resulting suspensions were drop-cast onto 300 mesh Cu grids coated with lacey carbon (Ted Pella) and allowed to dry (ca. 30 s) before being loaded into the microscope. Energy dispersive spectroscopy (EDS) was used to confirm the presence and location of cobalt particles. EDS maps were acquired using a dwell time of 20–50 μs. A beam shower technique (10 nA, 10 min) was used to reduce charging and contamination effects during EDS acquisition, as needed, and imaging was performed both prior and after beam showering to help factor out any effects of the beam shower on particle morphology or sintering behavior. Particle size measurements were conducted on STEM images using ImageJ software by manually measuring particle diameters of at least 100 particles for each sample. For nonspherical particles, the length (longest axis) and width (shortest axis) were measured, and the two values were averaged. For core–shell particles, the entire particle was measured, including the shell thickness. For the samples that instead formed lacy cobalt-containing networks, the diameter of the entire network was measured.

### Scanning Electron Microscopy (SEM)

For SEM analysis, postreaction samples were dispersed over carbon tape and analyzed with a Nova NanoSEM630 (FEI) operating at 15 kV acceleration voltage and a current of 5.6 nA. Energy dispersive spectroscopy (EDS) maps were acquired using an Ultim Max SSD EDS detector (Oxford Instruments) and analyzed using AZtec (Oxford Instruments) software.

### X-ray Photoelectron Spectroscopy (XPS)

XPS analysis was performed on a customized Physical Electronics VersaProbe III using monochromatic Al Kα radiation. Powder catalyst samples were transferred without air exposure into an argon-filled glovebox, pressed into carbon tape with a wooden “orange stick” to avoid metal contamination, and subsequently transferred into the XPS system using a PHI Mod. 07–111 K transfer vessel. Source-induced charging in raw data was observed, and energy scales were calibrated by assuming that the Si 2*p* envelope should appear at 103.5 eV. Wide range “survey” spectra were acquired with a pass energy of 280 eV, probed a region 100 μm in diameter, anode power of ∼23 W, and at normal photoelectron takeoff angle. High energy resolution spectra were acquired similarly but with a pass energy of 27 eV. To obtain sufficient *S*/*N* in high resolution cobalt 2*p* spectra, a large dwell time of 9 s/data point was used.

## Supporting Information Available

Surrogate model data collection; classification model information; Bayesian optimization data; HR-TEM and SAED data for optimized and unoptimized CoO nanoparticles; Rietveld refinement data; in situ XRD data for optimized, unoptimized, and incipient wetness impregnation supported CoO nanoparticles; XPS spectra of optimized, unoptimized, and incipient wetness impregnation supported CoO nanoparticles; STEM-HAADF images of reduced optimized and reduced unoptimized supported CoO nanoparticles with EDX mapping; and details of code (PDF)
