# Supplementary material for: Multivariate Bayesian Optimization of CoO Nanoparticles for CO2 Hydrogenation Catalysis
Source: J Am Chem Soc. 2024 May 10;146(20):14246–59. doi: 10.1021/jacs.4c03789 (PMC11117399; doi:10.1021/jacs.4c03789)
Supplement: Supplementary file 1 — ja4c03789_si_001.pdf [file ja4c03789_si_001.pdf]

SUPPORTING INFORMATION

Multivariate Bayesian Optimization of CoO Nanoparticles for CO_2 Hydrogenation Catalysis

Lanja R. Karadaghi,[‡a] Emily M. Williamson,[‡a] Anh T. To,[b] Allison P. Forsberg,[a] Kyle D. Crans,[a] Craig L. Perkins,[c] Steven C. Hayden,[c] Nicole J. LiBretto,[b] Frederick G. Baddour,[b] Daniel A. Ruddy,[b] Noah Malmstadt,[adef]* Susan E. Habas,[b]* and Richard L. Brutchey[a]*

[a] *Department of Chemistry, University of Southern California, Los Angeles, California 90089, United States*
[b] *Catalytic Carbon Transformation and Scale-Up Center, National Renewable Energy Laboratory, Golden, Colorado 80401, United States*
[c] *Materials Science Center, National Renewable Energy Laboratory, Golden, Colorado 80401, United States*
[d] *Mork Family Department of Chemical Engineering and Materials Science, University of Southern California, Los Angeles, California 90089, United States*
[e] *Department of Biomedical Engineering, University of Southern California, Los Angeles, California 90089, United States*
[f] *USC Norris Comprehensive Cancer Center, University of Southern California, 1441 Eastlake Ave, Los Angeles, California 90033, United States*

E-mails: malmstadt@usc.edu, susan.habas@nrel.gov, brutchey@usc.edu
‡These authors contributed equally.

Table of Contents

1. Surrogate Model Data Collection S2
   a) Response Characterization S2
   b) Screening Design S2
   c) Literature Mining S3
   d) Optimization Design S4
2. Classification Model S5
3. Bayesian Optimization S9
   a) Optimizing Size S11
   b) Optimizing Polydispersity S13
   c) Optimizing Shape Purity S15
   d) Multi-Response Optimization S17
   e) Additional Characterization S24
4. Code S28
5. References S24

1. Surrogate Model Data Collection

a) Response Characterization

The nanoparticle attributes that were mapped as a function of this experimental space include crystal phase, size, shape, and polydispersity. With the various experimental conditions from the surrogate model, we were able to access phase-pure rock salt CoO. Each observed crystal phase, or combination of phases, was assigned a unique code (**Table S1**). The crystal phase was assessed by powder X-ray diffraction (XRD), while size, shape, and polydispersity were each assessed by automated transmission electron microscopy (TEM) image analysis.

Table S1. Corresponding phases observed in the entire phase space for this system.

Phase No.	Indexed Phase(s)
1	no isolable product
2	amorphous cobalt oxide
3	fcc Co
4	Co_2C + fcc Co
5	rock salt CoO + fcc Co + Co_2C
6	rock salt CoO + fcc Co
7	rock salt CoO + wurtzite CoO + fcc Co
8	rock salt CoO
9	rock salt CoO + wurtzite CoO
10	wurtzite CoO
11	wurtzite CoO + fcc Co

The morphology of each nanoparticle product was quantified using an automated TEM image analysis pipeline, as previously reported by Williamson *et al.*[1] This pipeline assesses: (1) average nanoparticle size and (2) average nanoparticle size distribution. The shape purity was also assessed by this pipeline, which classified the morphology of each product into a statistically significant number of distinct shape groups. Nanoparticle ensembles classified into a smaller number of shape groups are more shape pure, so shape purity was optimized by minimizing the number of shape groups for each product.

b) Screening Design

The first step in the optimization was performing a parametric screening for the reaction space. The purpose of the screening was to systematically sample the reaction space to collect data on the experimental variables and their effects on four responses: nanoparticle size, size distribution of the ensemble (polydispersity), shape purity (# of shape groups), and crystal phase. The experimental variables investigated are: temperature (°C), oleylamine (OAm):$Co(acac)_2$ (mol/mol), oleic acid (OA):$Co(acac)_2$ (mol/mol), and hexadecanol:$Co(acac)_2$ (mol/mol). The boundary conditions for the experimental space are reported in **Table S2**. For this screening, the [Co] concentration is fixed at 50 mM and the reaction time is fixed at 1 h.

Table S2. Boundary Conditions for the Parametric Screening of the Synthesis of CoO Nanoparticles

**Experimental variable**	**High bound (+)**	**Low bound (–)**
Temperature (°C)	340	290
Oleylamine:$Co(acac)_2$ (mol/mol)	20	0.5
Oleic acid:$Co(acac)_2$ (mol/mol)	20	0.5
Hexadecanol:$Co(acac)_2$ (mol/mol)	6	0.5

With four experimental variables, a resolution V, 2^4 (2^N, where $N = 4$) full factorial screening design resulted in 19 total experiments (including one reaction at the center point and two replicates for statistical significance). Reaction conditions indicated by the screening matrices were coded for statistical analysis according to the methods described by Williamson *et al.*[2] The corresponding real values of each reaction performed in the screening and the observed responses are given in **Table S3.**

Table S3. Full-Factorial Reaction Screening Matrix and the Corresponding Responses.

**Temp.**	**OAm: $Co(acac)_2$**	**OA: $Co(acac)_2$**	**Hexadecanol: $Co(acac)_2$**	**Phase**	**Size**	**Polydispersity**	**Shape Purity**
(˚C)	*(mol/mol)*	*(mol/mol)*	*(mol/mol)*	*code*	*(nm)*	*(% SD)*	*# of shape groups*
290	20	20	0.5	8	61	60	6
340	20	20	6	7	25.2	99	5
340	20	0.5	0.5	4	17.9	46	5
290	0.5	0.5	0.5	10	--	--	--
290	20	0.5	6	6	45.5	58	12
290	0.5	20	6	1	13.8	45	4
340	0.5	0.5	6	5	27	99	5
340	0.5	20	0.5	2	24.6	42	11
315	10.25	10.25	3.25	6	--	--	--
290	20	20	6	8	21.3	42	4
290	0.5	0.5	6	7	34.5	100	8
340	0.5	20	6	2	47.6	63	5
340	20	0.5	6	8	57.3	59	5
290	20	0.5	0.5	4	17.3	46	5
290	0.5	20	0.5	2	44.3	95	10
340	0.5	0.5	0.5	10	--	--	--
340	20	20	0.5	6	21.1	34	5
340	0.5	0.5	0.5	10	45.3	100	4
340	20	20	0.5	6	65.9	100	5

c) Literature Data Mining

Additional reaction data was mined from the literature to increase the size of the surrogate model data set. Reactions that produced relevant phases of CoO nanoparticles within the set bounds of the investigation were added to the data set and are enumerated in **Table S4**. The difference in characterization techniques can also be assessed with this data, which show that the automated TEM image analysis pipeline used in this study is in good agreement with reported literature sizes. Interestingly, there are significant disparities between the reported polydispersity from the literature and the polydispersities reported by the automated TEM pipeline. Considering the similarities in reported size, this shows that the automated pipeline enables a much more thorough characterization of the polydispersity as it assesses the entire population of a product in comparison

to the polydispersities measured from a small sample of the population that are reported in the literature. For this reason, all morphological products from the literature were reassessed using the TEM morphology pipeline referenced in **Section a**.

Table S4. Reactions Mined from Literature and their Corresponding Responses[3–7]

Temp.	Time	OAm: $Co(acac)_2$	OA: $Co(acac)_2$	Hexadecanol :$Co(acac)_2$	Phase	Size	Lit. Size	Polydispersity	Lit. Polydispersity	Shape Purity	Ref.
(˚C)	*(min)*	*(mol/mol)*	*(mol/mol)*	*(mol/mol)*	*code*	*(nm)*	*(nm)*	*(% SD)*	*(% SD)*	*# of shape groups*	
200	60	50.4	0	0	10	44.2	38	48	10.5	5	3
200	60	100.3	0	0	10	28.3	26	48	15.4	6	3
200	60	200	0	0	10	27.3	21	42	19	4	3
205	180	30.7	0	0	8	22.4	6.2	46	9.3	4	4
250	120	30.7	0	0	8	17	17	57	11	5	4
205	30	30.7	0	0	8	9.8	4.5	84	6.7	7	4
230	60	30.7	0	0	8	--	--	--	--	--	4
250	60	30.7	0	0	8	--	--	--	--	--	4
210	60	2.0	0.5	1.5	8	9.3	8	28	10	4	5
210	60	2.0	0.5	1.5	8	12.4	13	24	10	3	5
240	60	2.0	0.5	1.5	8	13	20	29	--	5	5
240	60	2.0	0.5	1.5	8	14.5	25	39	--	5	5
300	60	2.0	0.5	1.5	8	34.5	25	29	--	4	6
210	60	2.0	0.5	1.5	8	16.8	8	31	--	4	6
250	60	15.0	0	0	8	42.8	8	48	--	11	7
250	60	7.5	0	0	8	30.7	9	63	--	5	7
250	60	5.0	0	0	8	15	11	57	--	4	7
250	60	3.7	0	0	8	16	13	43	--	5	7
250	60	3.0	0	0	8	17.5	15	38	--	4	7
200	60	3.0	0	0	8	16.2	11	100	--	7	7
300	60	3.0	0	0	8	23.2	38	49	--	4	7

d) Optimization Design

After the full-factorial screening design was performed, the reaction time was added as an additional variable and reactions from a Doehlert optimization matrix for five variables were performed to increase the number of data points and the statistical significance. Time was added to the investigation due to its apparent significance on observed responses seen in the reactions mined from the literature. The bounds of the study were also expanded for this reason, according to **Table 1** in the main text. All reactions performed to construct the surrogate model were re-coded according to the new bounds as per the method described by Williamson *et al.*[2] The real values of the reaction conditions and the resulting responses of each reaction are given in **Table S5**.

Table S5. Reactions Indicated by a Doehlert Matrix for Five Variables and their Corresponding Responses

Temp.	Time	OAm: $Co(acac)_2$	OA: $Co(acac)_2$	Hexadecanol: $Co(acac)_2$	Phase	Size	Polydispersity	Shape Purity
(°C)	*(min)*	*(mol/mol)*	*(mol/mol)*	*(mol/mol)*	*code*	*(nm)*	*(%SD)*	*# of shape groups*
240	105	21.35	5	2.39	8	20	56	4
240	105	21.35	15	2.39	8	18.5	56	4
307	105	21.35	10	2.39	8	18.8	51	9
263	105	21.35	10	4.84	6	17.2	48	5
263	163.2	37.11	10	3.0	6	15.7	38	4
263	105	100.25	10	3.0	3	66.3	63	7
195	105	100.25	5	3.0	10	14.2	44	4
285	163.2	116.01	15	3.61	6	12.6	66	11
263	105	100.25	0	3.0	9	18.6	37	4
195	46.8	84.49	5	2.39	6	14.7	47	8
263	105	100.25	20	3.0	3	21	97	10
330	105	100.25	5	3.0	6	13.6	44	9
202	45.3	11.89	0	1.26	8	14.5	43	4
190	154.1	53.22	15	4.76	8	14.1	51	4
281	32.6	114.25	20	0.11	6	15.8	69	6
185	88.3	200	0.9	0.48	8	15.3	48	6
243	59.3	149.56	15	3.10	8	16.4	52	5
191	48.2	200	0	0.0	9	22.5	27	4
240	179	103.93	15	1.80	6	16.8	57	8
197	119.8	117.97	5	3.70	11	13.7	55	4
185	70.5	199.99	0	1.23	8	15	36	4
255	31.8	199.03	10	2.10	8	23.5	45	6
224	67.5	0.5	0	1.50	6	14.1	48	4
208	49	9.73	0	1.24	8	15.6	51	9
211	60	1.93	0	1.51	9	15.5	46	5
191	47.8	199.99	0	0.0	10	15.2	42	4
185	30	0.5	0	0.0	8	12.8	51	9
204	71.3	100.25	1	0.15	6	13.3	38	6
340	180	200	20	6.0	3	81.9	83	6
263	105	100.25	10	3.0	6	10.7	42	5
301	60	0.5	0	1.8	6	16.6	41	5

2. Classification Model

The classification model was trained in MATLAB using the 72 reactions in the surrogate model, with an ensemble of trees using the *templateTree* function with the 'Surrogate' hyperparameter set to 'on' using the bag method over 500 learning cycles, resulting in an initial training data set of 36,000 observations (72×500). Specifically, bootstrap aggregation, or bagging with random predictor selections at each split (random forest), was used as it was predicted to be best for the multiclass nature of the data after a Bayesian optimization of the hyperparameters. The classification bagged ensemble model was trained with leave-one-out cross-validation. This evaluation method was chosen as it is ideal for smaller datasets, providing a much less biased

measure of test error compared to using a single test set because we repeatedly fit a model to a dataset that contains *n*–1 observations.[8] The classification accuracy of the initial surrogate model was 93.4%, misclassifying only one out of the 16 reactions in the testing set (**Figure S1**). After the nine iterations of the Bayesian optimization, the predictions accuracy improved to 99%, with a re-substitution loss of 0.0097, which equates to the misclassification rate. The closer the model predictions are to the observations, the smaller the misclassification error will be, and error ≤ 0.05 is considered acceptable, rendering our model statistically significant.[8]

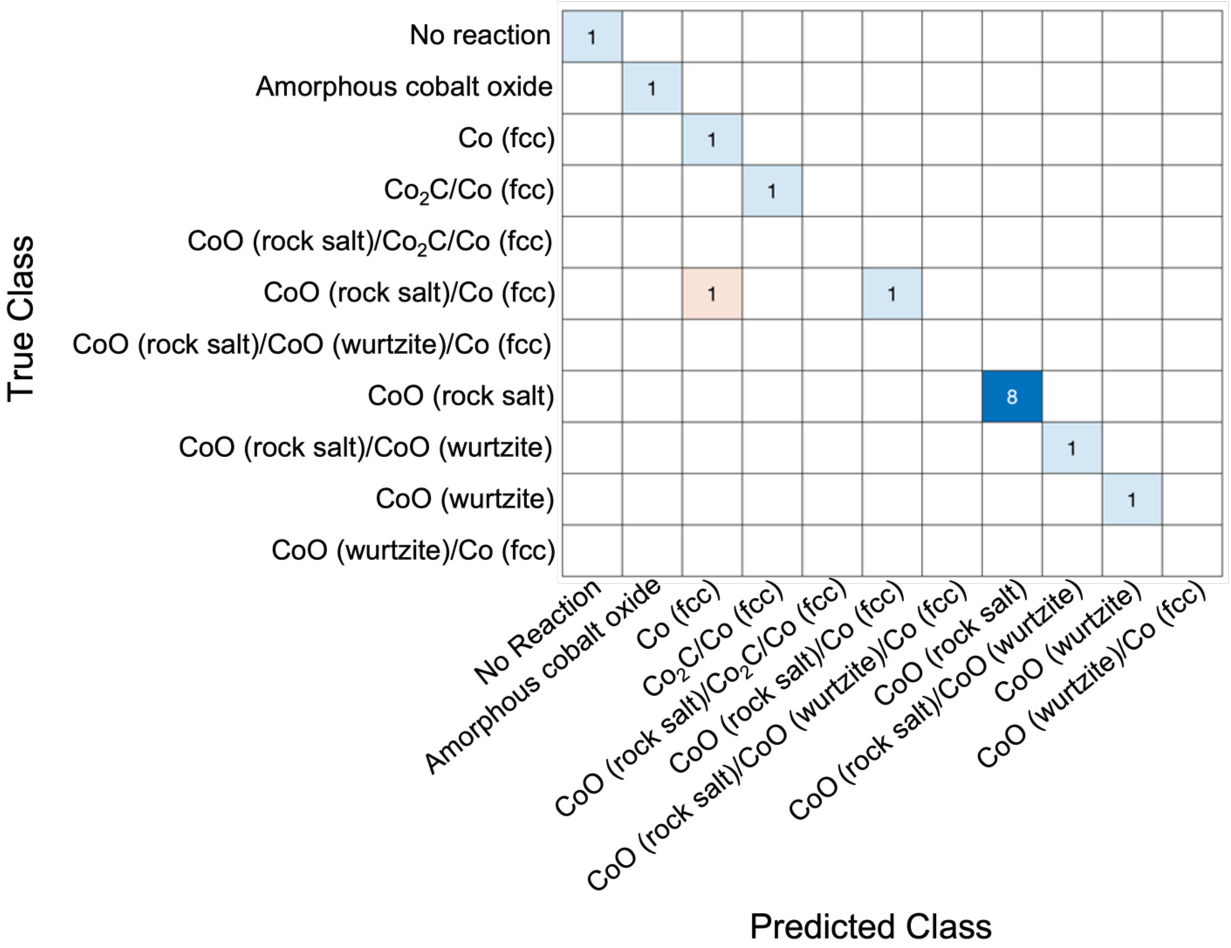

Figure S1. Confusion chart of the classification model predictions, showing correct predictions in blue and incorrect predictions in orange.

Univariate feature ranking for classification (*fsschi2* function) ranks features (predictors) using chi-squared tests. The predictor variables and the response variable (phase) from the training data were provided to a function that returns the indices of predictors ordered by predictor importance. This means the first predictor returned is the most important predictor. The feature rankings are illustrated in **Figure 1** in the main text and patterns in the surrogate model data illustrating the relationship between input variable and phase can be seen in **Figure S2**.

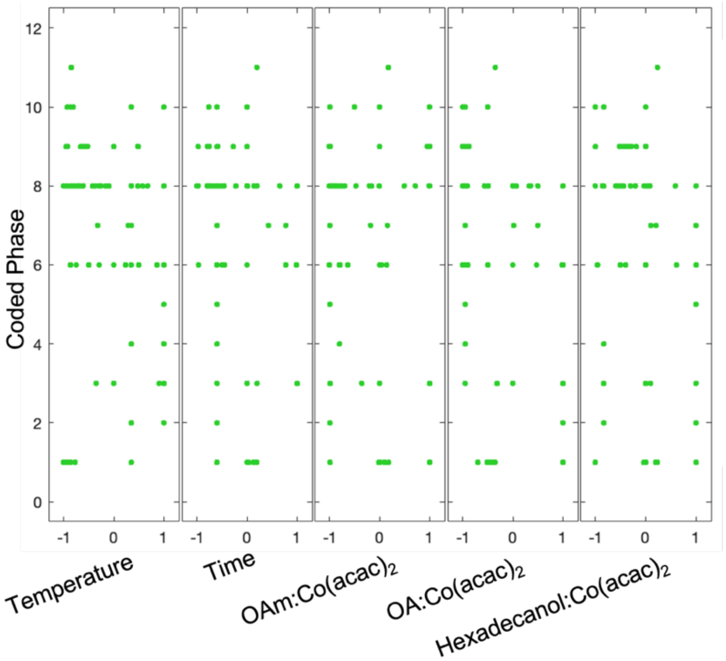

Figure S2. The patterns of the coded values for each input variable (x-axis) relating to each specific phase (y-axis).

The surrogate model data is visualized in 3-dimensions using classic multidimensional scaling (CMD) to compress the 5-dimensional data into 3-dimensions using the first three principal components (PCs) (**Figure S3a,b**). *ScatteredInterpolant* was used to interpolate the 3-D dataset of scattered surrogate model data. This returns the interpolant (F) for the experimental dataset. Interpolant F can be evaluated at a set of query points, such as (xq, yq, zq) in 3-D, to produce predicted interpolated values $vq = F(xq, yq, vq)$. The resulting phase map for the three most significant experimental variables is shown in **Figure 1** and the phase map of the entire data scaled down to 3-dimensions is shown in **Figure S3c**.

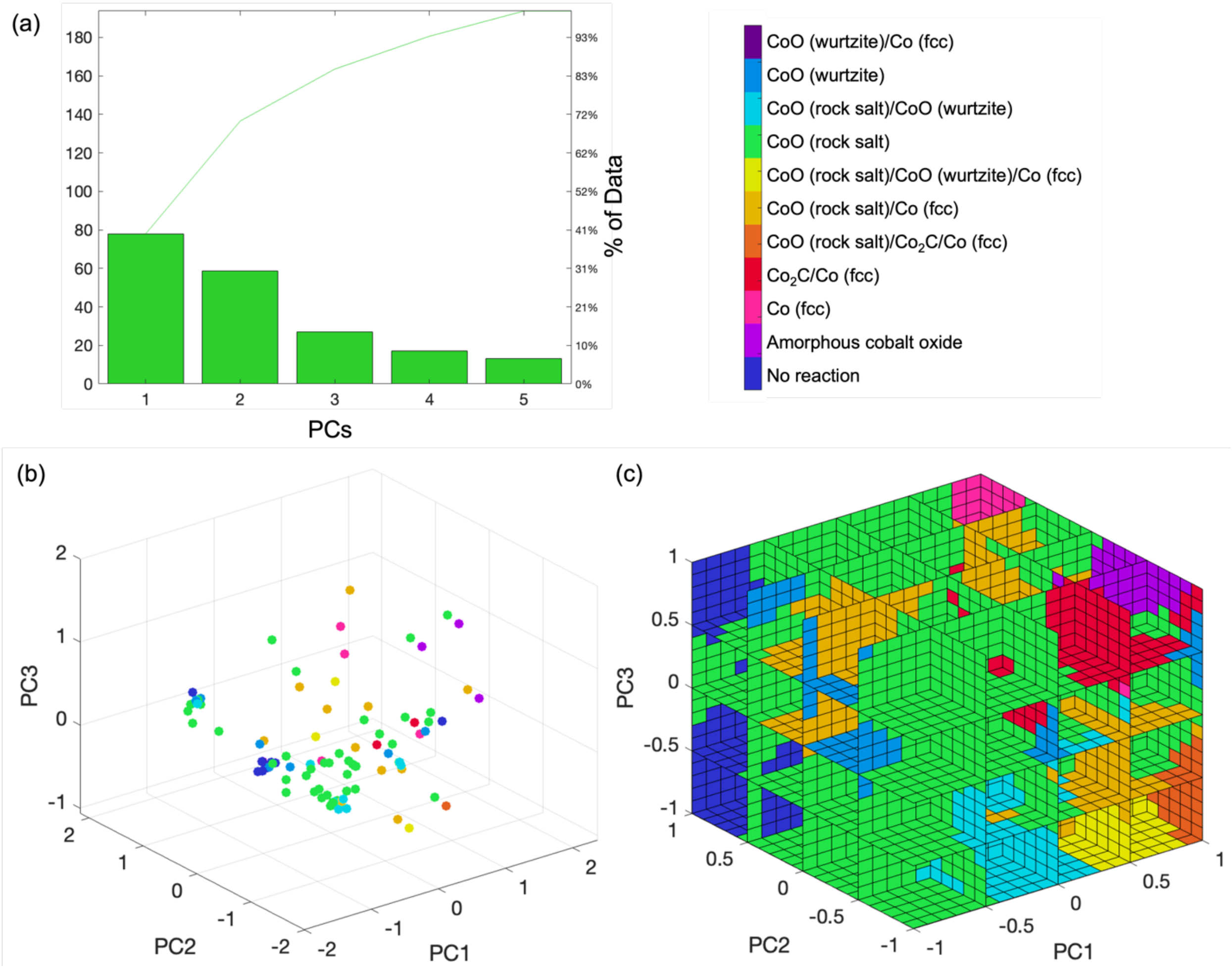

Figure S3. (a) Pareto chart of the principal components (PCs) after the classic multidimensional scaling. The first three principal components describe approximately 88% of the data and are used to create the scatterplot in (b) and the interpolation of the scatterplot in (c). Phases correspond to the colors in the legend.

3. Bayesian Optimization

Individual iterative Bayesian optimizations were run for three of the responses: nanoparticle size, polydispersity, and shape purity. The Bayesian optimizations were adapted from the methods described by Frey and coworkers for a chemically informed data-driven optimization (ChIDDO), with the trained classification model incorporated into each Bayesian model to predict crystal phase at each iteration of the optimization.[9] The surrogate models for each response were created using regression-based response surface methodology (RSM), as depicted in **Figure 2** in the main text and previously described by Williamson *et al.*[2]

An acquisition function uses the current information, X^{exp} and y^{exp}, and the surrogate model predictions to calculate how informative a possible design condition is expected to be based on the criteria for the respective acquisition function.[9] To determine the most informative design point to sample next, two acquisition functions – expected improvement (EI) and modified rank-batch (MRB) – were tested to find a local maximum of the acquisition function score. This process was repeated 30 times at 21 different initiation points to get closer to the global solution for each response. The design point with the maximum score that was also predicted to produce the target rock salt phase was subsequently added to x^{next}. Two acquisition functions, EI and MRB, were tested:

The EI acquisition function is given by **eq. S1**:

$$EI(x) = (\mu(x) - f(x^{-}) - \xi)\psi\left(\frac{\mu(x) - f(x^{-}) - \xi}{\sigma(x)}\right) + \sigma(x)\phi\left(\frac{\mu(x) - f(x^{-}) - \xi}{\sigma(x)}\right) \quad \textbf{(S1)}$$

where $\mu(x)$ is the mean of the regressor at x, $\sigma(x)$ is the variance of the regressor at x, f is the function to be minimized, x^{-} is the location of the estimated minimum, ξ is the exploration/exploitation parameter, $\psi(z)$ is the cumulative distribution function of a standard Gaussian distribution, and $\phi(z)$ is the density function of a standard Gaussian distribution.[9]

The MRB mode sampling function was developed by Frey *et al.* from the work of Cardoso *et al.*[9,10] The MRB acquisition function calculates a score consisting of three normalized parameters: a distance score (Δ), an uncertainty score (Γ), and the objective function prediction (Ω). The distance score was calculated as:

$$\Delta = 1 - 1/\left(1 + min\sqrt{\sum_{i=1}^{d}\left(x_i - x_i^{exp}\right)^2}\right) \quad \textbf{(S2)}$$

Where $\sqrt{\sum_{i=1}^{d}\left(x_i - x_i^{exp}\right)^2}$ is the minimum distance between the proposed set of conditions, x, and each of the known sets of conditions, x^{exp}. The uncertainty score (Γ) is the standard deviation of the RSM prediction at x normalized compared to the maximum and minimum observed standard deviation. The objective function prediction (Ω) is y^{pred} at x normalized compared to the maximum and minimum observed prediction.

The score that is calculated at each step in the minimization process for the respective x is:

$$Score = \beta\Delta + \beta\Gamma + \Omega \quad \textbf{(S3)}$$

where β is a tradeoff value. A high value of β encourages more exploration; that is, encourages searching unknown areas of the design space. A lower value of β encourages exploitation; that is, searching locally near the current maximum prediction. All the acquisition functions include a tradeoff value that decreases as more experiments are run, moving from exploration to exploitation. For MRB, β changes linearly from 1 to 0. For EI, β changes logarithmically from around 0.05 to 1×10^{-7}.

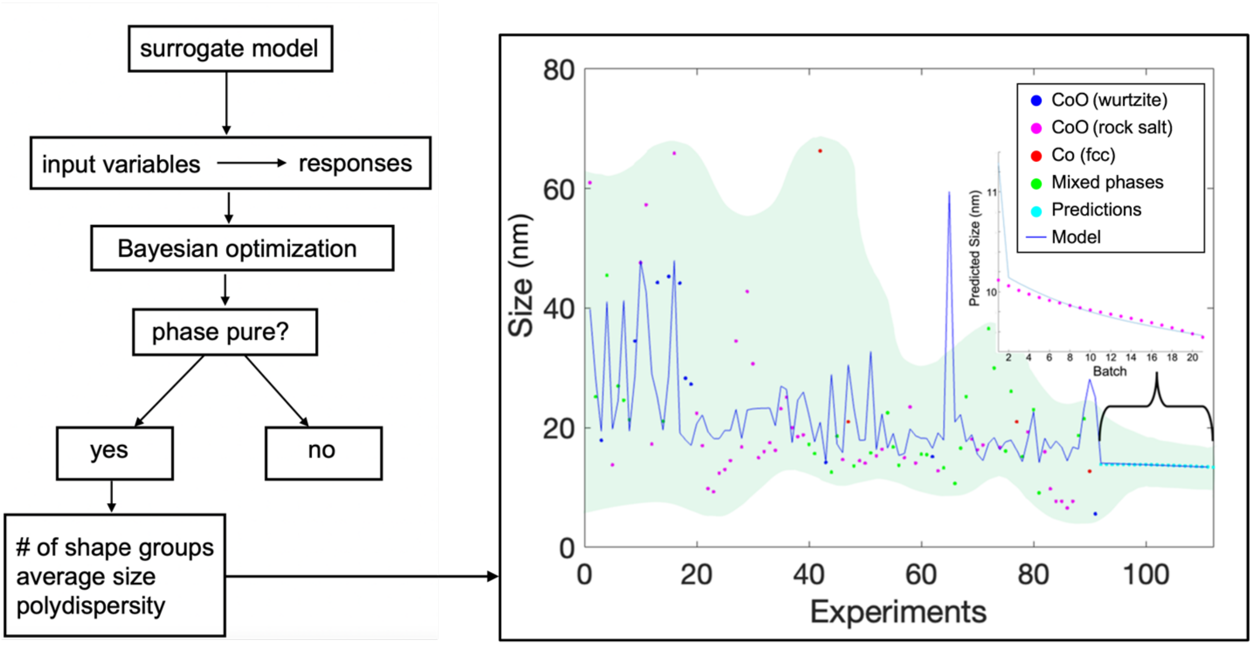

Scheme S1. High-level depiction of the Bayesian optimization flow scheme. Final graph depicts Bayesian optimization to minimize size, with the crystal phase of each experiment indicated by color according to the legend. Predicted size distribution (*i.e.*, polydispersity) is shown in green. The model predictions after training are shown in teal, with the phase shown in the inset graph.

The last step in **Scheme S1** can be described as follows:

Input: A set of N_{init} experimental points, X^{exp}, where $X^{exp} \in_R$ parameter space (PS), evaluated to give the objective function value, y^{exp}. $P^{exp} = \{X^{exp}, y^{exp}\}$.

1. P^{exp} is passed to the RSM to make predictions, μ^{RSM}, and uncertainties, σ^{RSM}, at any point in PS.
2. P^{exp}, μ^{RSM}, and σ^{RSM} are passed to the acquisition function to select n_b new experiments, X^{next}, to evaluate.
3. Trained classifier predicts resultant phase of X^{next}.
4. Condition *phase*(X^{next}) = target phase is evaluated as a binary (1) or (0).
5. If *phase*(1), \objective function is evaluated at X^{next} to give y^{next} (Pnext) and then $P^{exp} = P^{exp} \cup P^{next}$.
6. Steps 1-3 repeat until 30, N_{total}, experiments are evaluated.
7. Return: P^{exp}.

a) Optimizing Nanoparticle Size

$$Surrogate\ Model\ for\ Size \\ = 21.053 + 24.8994(Temperature) - 7.10049(Time) \\ + 5.42007(OAm{:}\,Co(acac)_2) - 7.5423(OA{:}\,Co(acac)_2) \\ + 14.7241(Hexadecanol{:}\,Co(acac)_2) + 0.989089(Temperature^2) \\ - 26.4194(Temperature \times Time\ ) \\ + 17.052(Temperature \times OAm{:}\,Co(acac)_2) \\ - 1.06488(Temperature \times OA{:}\,Co(acac)_2) \\ + 20.3503T(emperature \times Hexadecanol{:}\,Co(acac)_2) - 4.52205(Time^2) \\ + 3.03148(Time \times OAm{:}\,Co(acac)_2) - 32.0434(Time \times OA{:}\,Co(acac)_2) \\ + 38.0946(Time \times Hexadecanol{:}\,Co(acac)_2) \\ + 0.859547(OAm{:}\,Co(acac)_2^2) \\ - 17.4581(OAm{:}\,Co(acac)_2 \times OA{:}\,Co(acac)_2) \\ + 9.13135(OAm{:}\,Co(acac)_2 \times Hexadecanol{:}\,Co(acac)_2) \\ + 21.846(OA{:}\,Co(acac)_2^2) \\ + 14.4873(OA{:}\,Co(acac)_2 \times Hexadecanol{:}\,Co(acac)_2) \\ - 23.1916(Hexadecanol{:}\,Co(acac)_2^2)$$

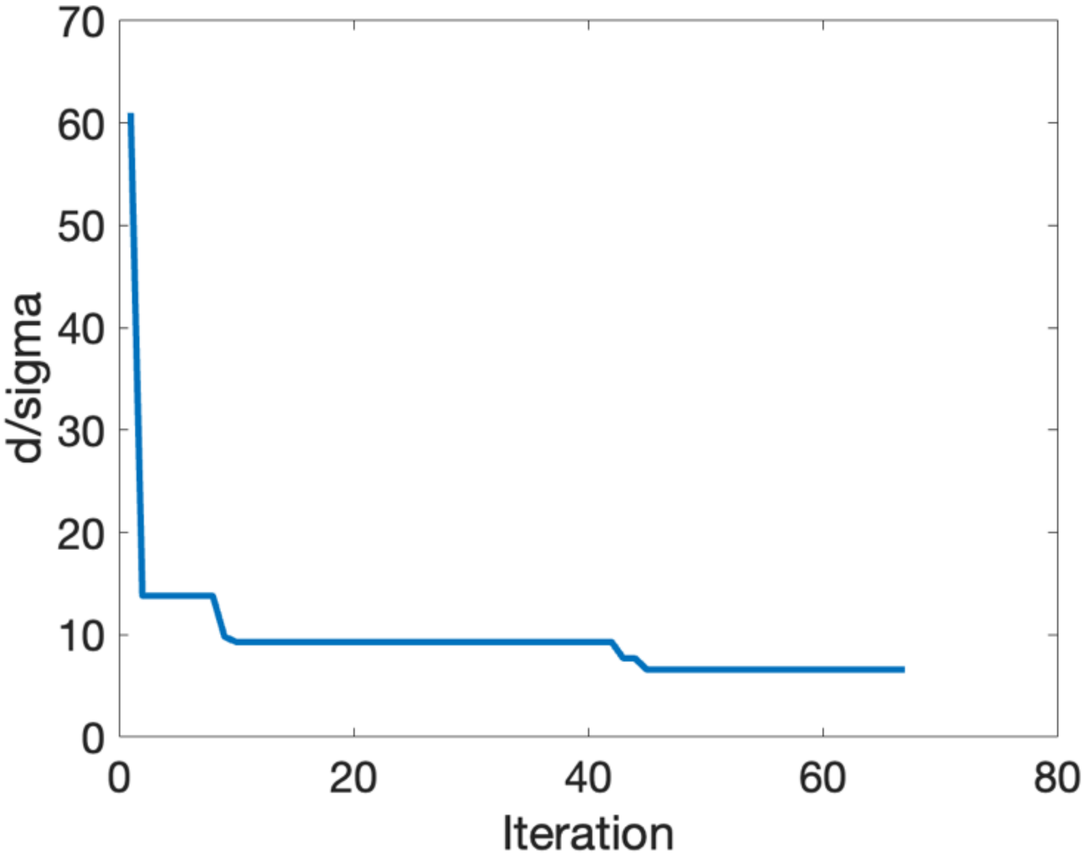

Figure S4. The average of the results for the Bayesian optimization of nanoparticle size.

Acquisition function: ***EI***

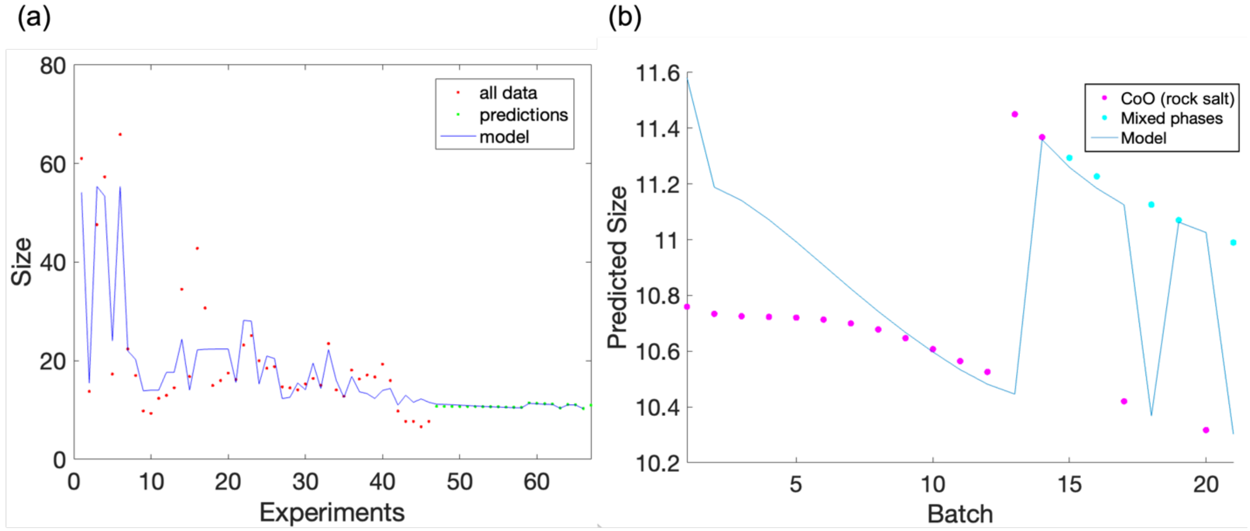

Figure S5. (a) Bayesian optimization to minimize nanoparticle size using the EI acquisition function, with predicted size after training shown in green. (b) Magnification of the model predictions indicated by green circles in part (a) with the phase corresponding to the legend.

Acquisition function: ***MRB***

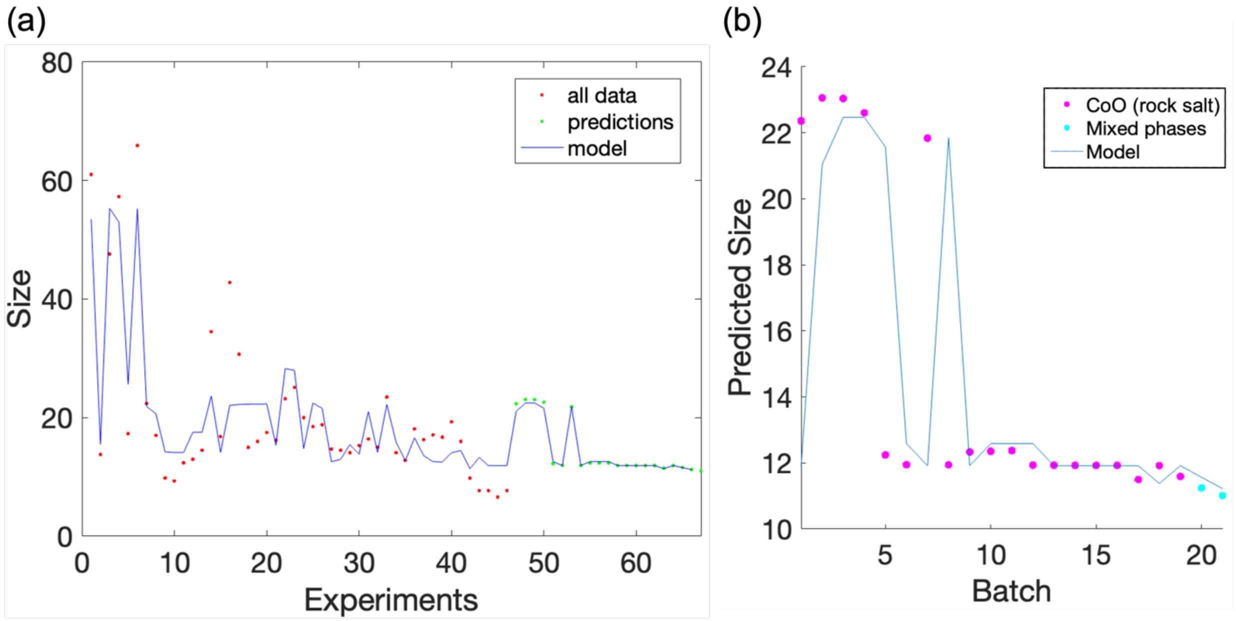

Figure S6. (a) Bayesian optimization to minimize nanoparticle size using the MRB acquisition function, with predicted size after training shown in green. (b) Magnification of the model predictions indicated by green circles in part (a) with the phase corresponding to the legend.

b) Optimizing Nanoparticle Polydispersity

$$\begin{aligned} &Surrogate\ Model\ for\ Polydispersity \\ &= 59.7 + 22.7(Temperature) - 27.5(Time) - 9.7(OAm{:}\,Co(acac)_2) \\ &+ 26.5(OA{:}\,Co(acac)_2) - 8.7(Hexadecanol{:}\,Co(acac)_2) \\ &+ 20.6(Temperature^2) - 44.9(Temperature \times Time) \\ &+ 19.8(Temperature \times OAm{:}\,Co(acac)_2) \\ &+ 11.5(Temperature \times OA{:}\,Co(acac)_2) \\ &+ 51.0(Temperature \times Hexadecanol{:}\,Co(acac)_2) - 47.7(Time^2) \\ &- 38.1(Time \times OAm{:}\,Co(acac)_2) - 0.8(Time \times OA{:}\,Co(acac)_2) \\ &+ 43.1(Time \times Hexadecanol{:}\,Co(acac)_2) - 34.7(OAm{:}\,Co(acac)_2^2) \\ &+ 12.2(OAm{:}\,Co(acac)_2 \times OA{:}\,Co(acac)_2) \\ &- 0.9(OAm{:}\,Co(acac)_2 \times Hexadecanol{:}\,Co(acac)_2) \\ &+ 14.0(OA{:}\,Co(acac)_2^2) + 19.9(OA{:}\,Co(acac)_2 \times Hexadecanol{:}\,Co(acac)_2) \\ &- 19.5(Hexadecanol{:}\,Co(acac)_2^2) \end{aligned}$$

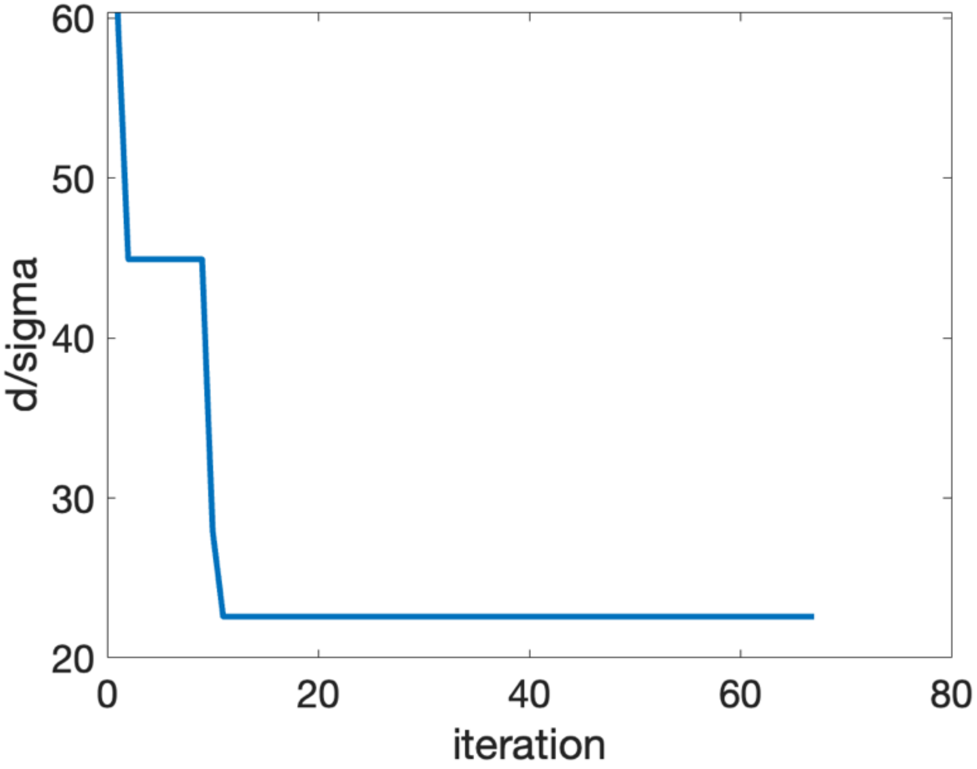

Figure S7. The average of the results for the Bayesian optimization of nanoparticle polydispersity.

Acquisition function: ***EI***

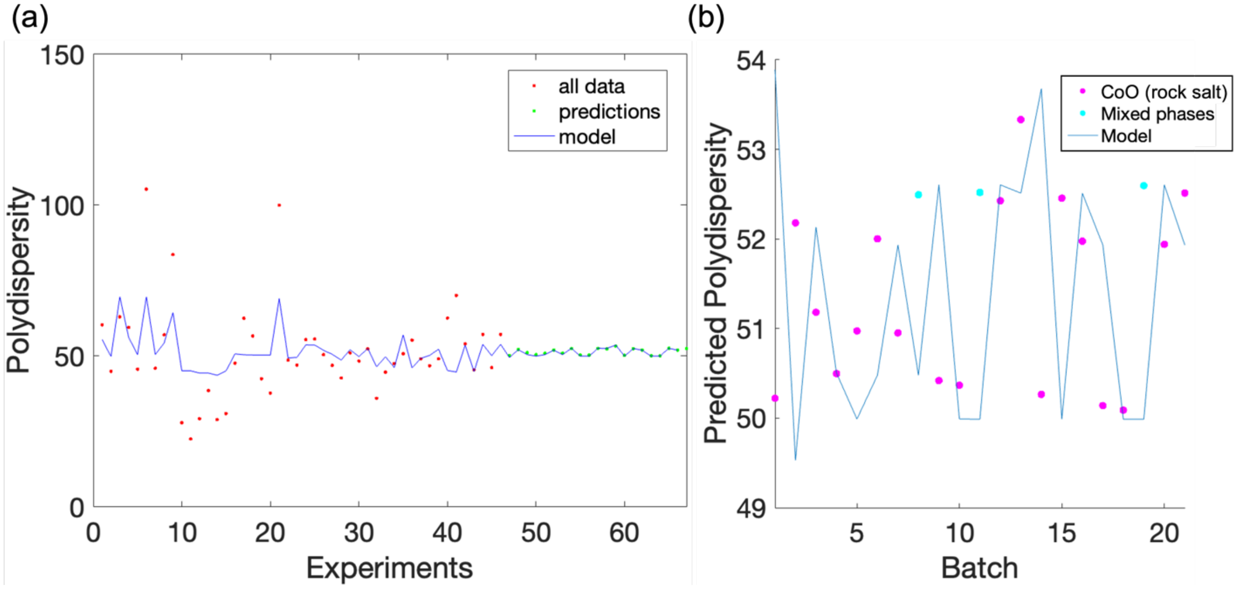

Figure S8. (a) Bayesian optimization to minimize nanoparticle polydispersity using the EI acquisition function, with predicted polydispersity after training shown in green. (b) Magnification of the model predictions indicated by green circles in part (a) with the phase corresponding to the legend.

Acquisition function: ***MRB***

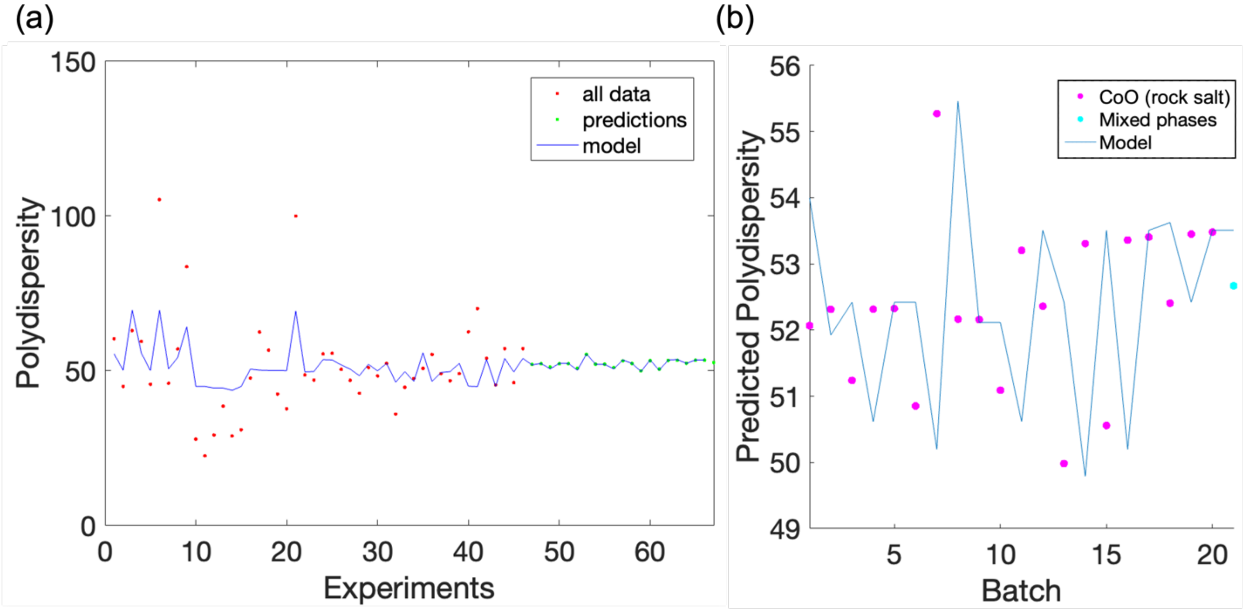

Figure S9. (a) Bayesian optimization to minimize nanoparticle polydispersity using the MRB acquisition function, with predicted polydispersity after training shown in green. (b) Magnification of the model predictions indicated by green circles in part (a) with the phase corresponding to the legend.

c) Optimizing Nanoparticle Shape Purity

$$Surrogate\ Model\ for\ Shape\ Purity = 14.7 + 13.7(Temperature) + 5.3(Time) + 8.5(OAm{:}\,Co(acac)_2) - 0.8(OA{:}\,Co(acac)_2) - 2.9(Hexadecanol{:}\,Co(acac)_2) + 1.1(Temperature^2) - 4.7(Temperature \times Time) + 10.9(Temperature \times OAm{:}\,Co(acac)_2) + 4.0(Temperature \times OA{:}\,Co(acac)_2) + 3.6(Temperature \times Hexadecanol{:}\,Co(acac)_2) - 1.9(Time^2) + 6.4(Time \times OAm{:}\,Co(acac)_2) + 2.1(Time \times OA{:}\,Co(acac)_2) + 4.6(Time \times Hexadecanol{:}\,Co(acac)_2) - 3.3(OAm{:}\,Co(acac)_2^2) - 2.3(OAm{:}\,Co(acac)_2 \times OA{:}\,Co(acac)_2) - 2.9(OAm{:}\,Co(acac)_2 \times Hexadecanol{:}\,Co(acac)_2) - 1.5(OA{:}\,Co(acac)_2^2) + 3.0(OA{:}\,Co(acac)_2 \times Hexadecanol{:}\,Co(acac)_2) - 2.5(Hexadecanol{:}\,Co(acac)_2^2)$$

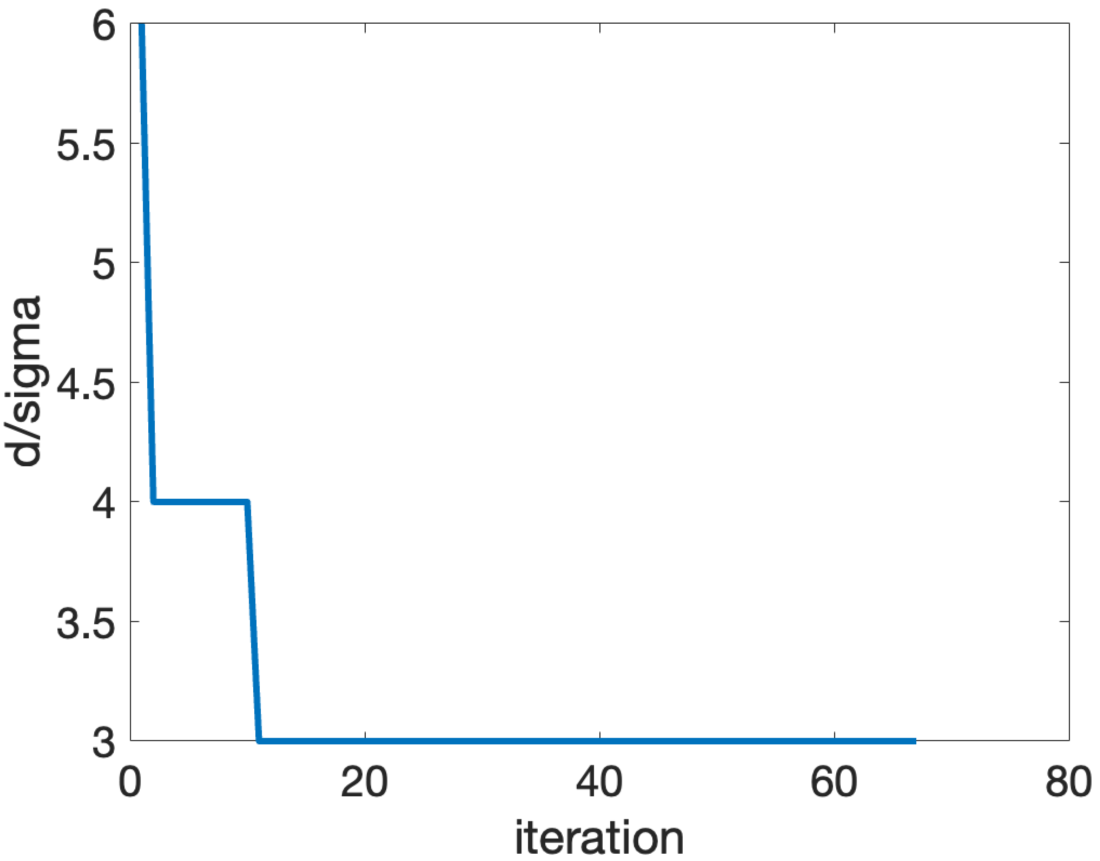

Figure S10. The average of the results for the Bayesian optimization of nanoparticle shape purity.

Acquisition function: ***EI***

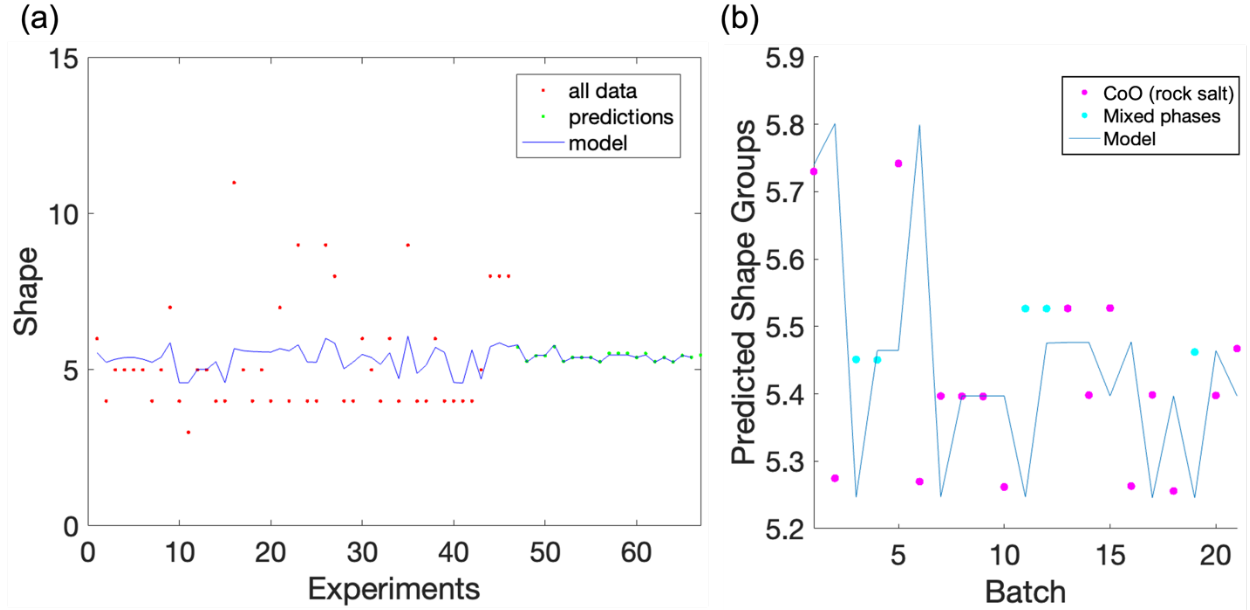

Figure S11. (a) Bayesian optimization to minimize the number of nanoparticle shape groups using the EI acquisition function, with predicted shape groups after training shown in green. (b) Magnification of the model predictions indicated by green circles in part (a) with the phase corresponding to the legend.

Acquisition function: ***MRB***

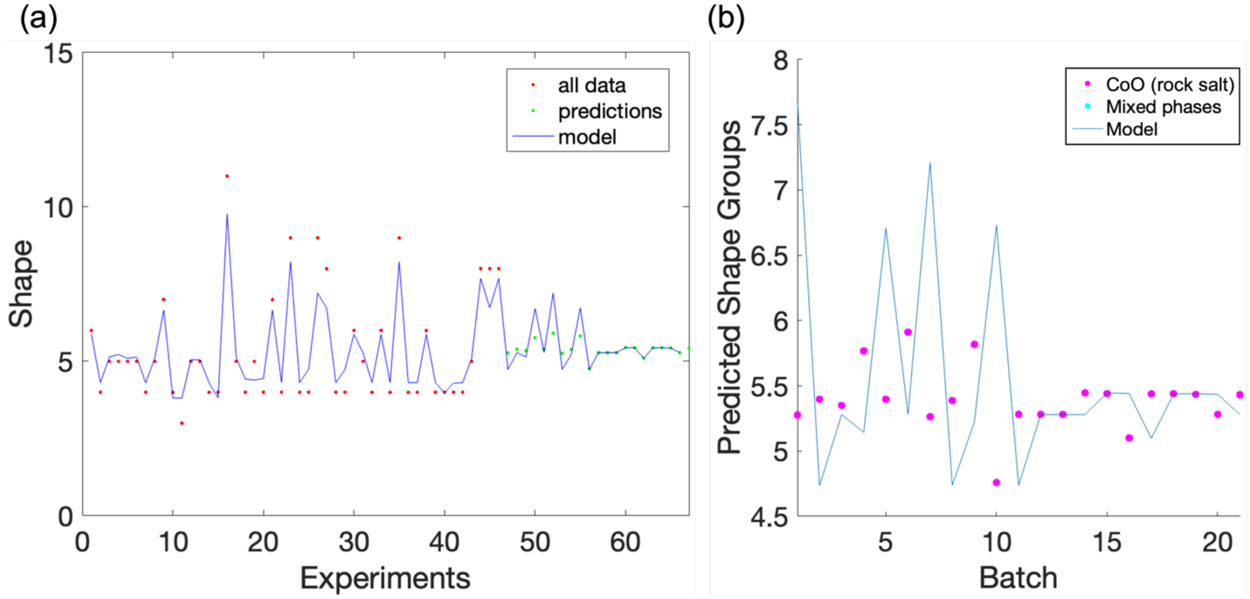

Figure S12. (a) Bayesian optimization to minimize the number of nanoparticle shape groups using the MRB acquisition function, with predicted shape groups after training shown in green. (b) Magnification of the model predictions indicated by green circles in part (a) with the resultant phase corresponding to the legend.

d) Multi-Response Optimization

The models from each individual Bayesian optimization were compiled into a single model using a desirability function, as previously described by Williamson *et al.*[2] This model was use to maximize desirability for a multi-objective optimization of all three responses. After nine iterations of the Bayesian optimization, the reactions shown in **Table S6** were added to the fitness function. The full parameter space is visualized in **Figure S14**.

Table S6. Reaction Conditions Predicted from the Bayesian Optimization with the Resulting Responses

**Temp.**	**Time**	**OAm: $Co(acac)_2$**	**OA: $Co(acac)_2$**	**Hexadecanol: $Co(acac)_2$**	**Phase**	**Size**	**Shape Purity**	**Polydispersity**
(˚C)	*(min)*	*(mol/mol)*	*(mol/mol)*	*(mol/mol)*	*code*	*(nm)*	*# of shape groups*	*(% SD)*
222	61.1	1.0	0.9	2.4	9	25.2	11	58
234	66.7	7.7	0.5	1.6	8	18.1	4	55
196	105.0	100.3	5.7	3.0	1	--	--	--
196	62.1	171.5	0.0	1.3	8	16.3	4	49
205	115.4	80.0	4.3	3.2	8	17.1	6	47
217	84.3	0.65	1.0	2.1	9	36.6	10	122
213	31.8	0.6	0.9	1.8	9	30.0	7	85
196	51.4	200.0	0.0	0.4	8	16.7	4	49
238	137.1	82.5	0.0	3.3	7	16.1	5	41
215	60.0	0.6	1.2	2.0	9	26.1	4	101
235	120.0	64.7	6.9	3.3	3	21.0	6	131
195	105.0	100.3	5.0	3.0	6	15.2	4	49
209	59.9	1.9	0.5	1.5	8	19.3	4	63
300	1.0	0.5	0.3	1.6	9	23.0	5	72
191	106.2	101.1	4.9	2.9	10	9.1	4	52
215	1.0	2.6	0.6	1.6	8	15.98	4	70
185	54.1	200.0	0.0	0.0	8	9.8	4	54
206	54.0	3.8	0.4	1.7	8	7.7	5	45
193	105.0	200.0	3.0	0.0	1	--	--	--
193	105.0	100.3	5.0	3.0	1	--	--	--
202	105.7	98.8	5.1	2.9	1	--	--	--
200	105.5	99.6	5.0	2.9	8	6.6	8	46
186	107.5	98.6	4.9	2.8	1	--	--	--
191	106.2	101.6	5.0	3.0	1	--	--	--
194	105.4	99.4	4.9	2.9	1	--	--	--
195	53.9	200.0	0.0	0.0	8	7.7	8	57
189	45.7	195.2	0.0	0.1	8	7.8	8	56
300	60.0	1.1	0.5	1.4	9	21.5	10	71
333	60.0	1.9	0.5	0.5	3	12.7	4	48
196	118.2	118.3	6.5	3.7	1	--	--	--
197	60.0	118.0	6.5	3.7	1	--	--	--
290	60.0	0.1	0.1	0.1	10	9.9	4	45

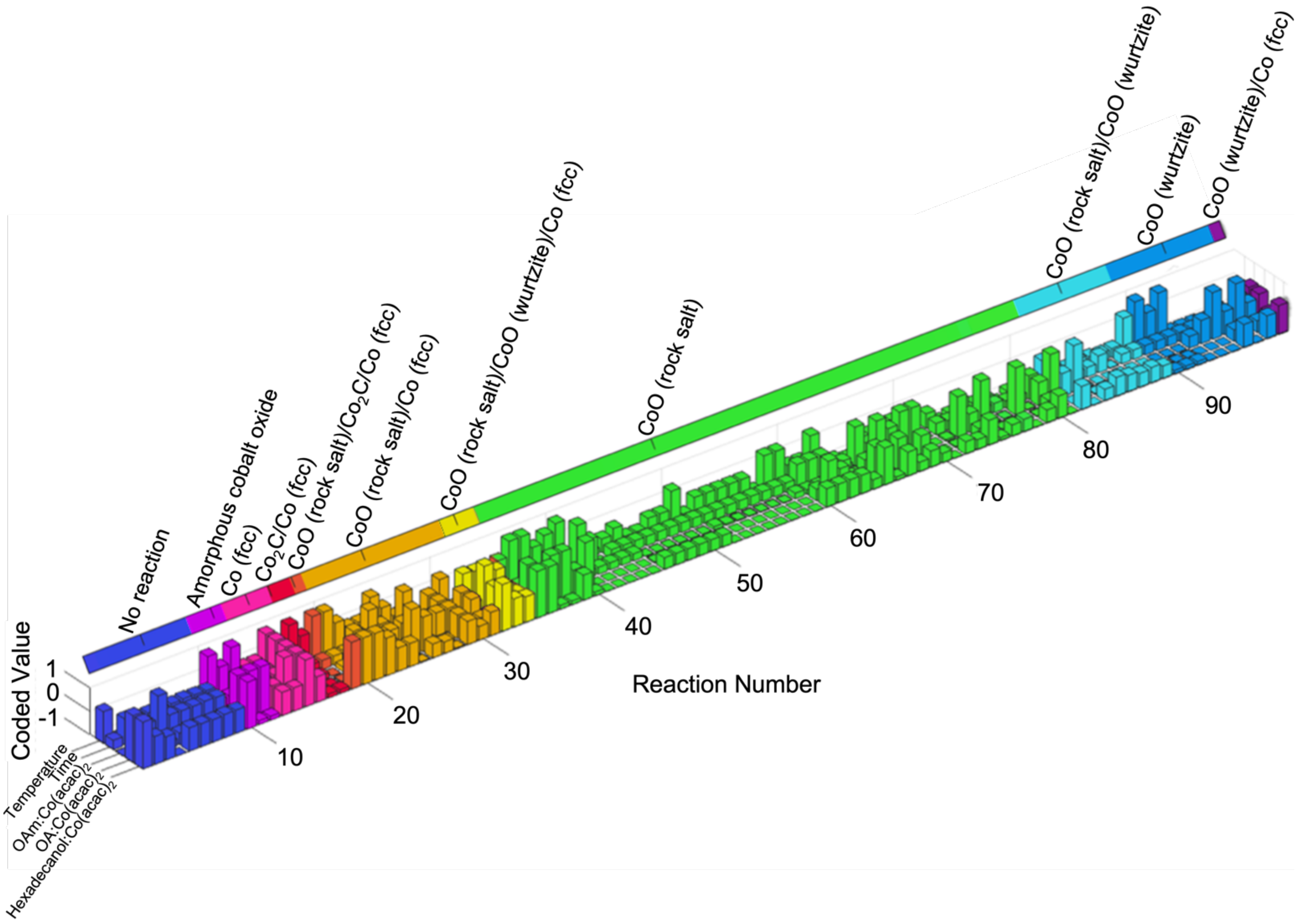

Figure S13. A visualization of the entire phase space with each reaction condition depicted as a bar plot and the resulting phases color coded.

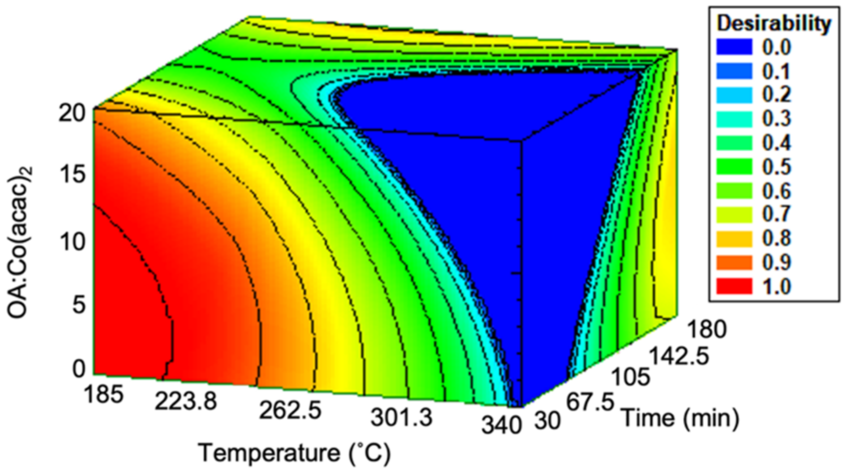

Figure S14. Overall desirability plot for size, polydispersity, and shape as a function of the three most significant experimental variables. A desirability score of 1 corresponds to each of the predicted optima in the multiple response optimization being identical to their individual optima (100% desirability).

e) Additional Characterization

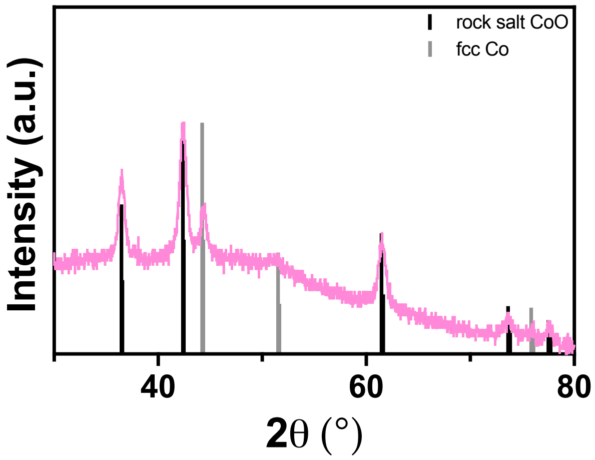

Figure S15. Powder XRD pattern of a two-phase mixture of rock salt CoO and metallic fcc Co produced by a reaction with a OA:$Co(acac)_2$ ratio of 10, a reaction time of 110 min, a temperature of 263 ˚C, and ratios of OAm:$Co(acac)_2$ and hexadecanol:$Co(acac)_2$ of 25 and 4, respectively.

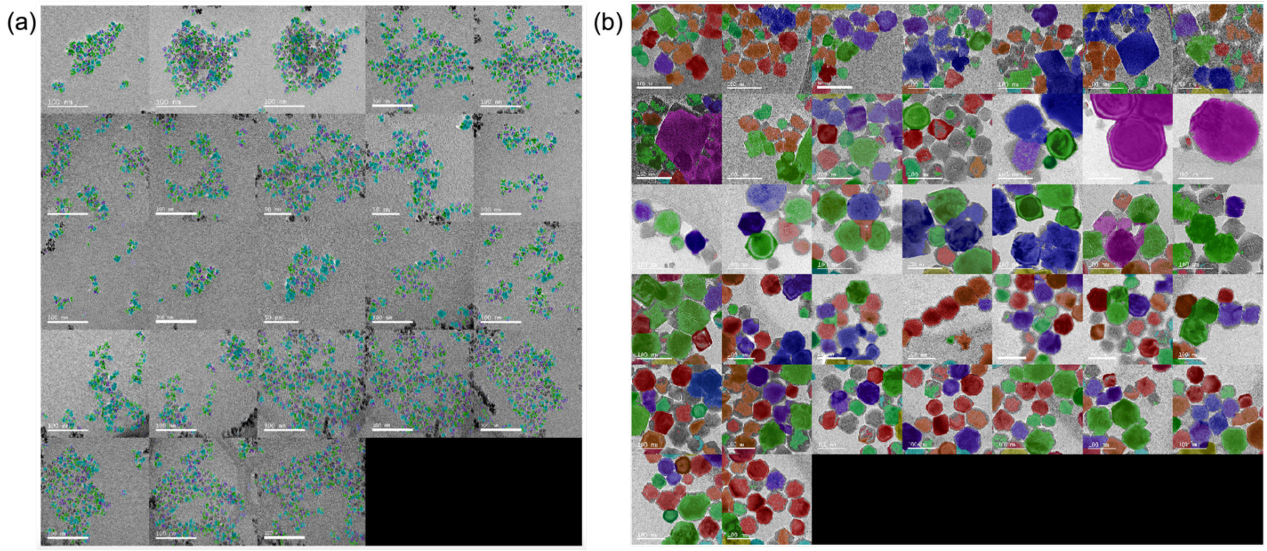

Figure S16. Shape group labels for (a) the ensemble of optimized rock salt CoO nanoparticles and (b) the ensemble of unoptimized rock salt CoO nanoparticles taken from automated TEM image analysis.

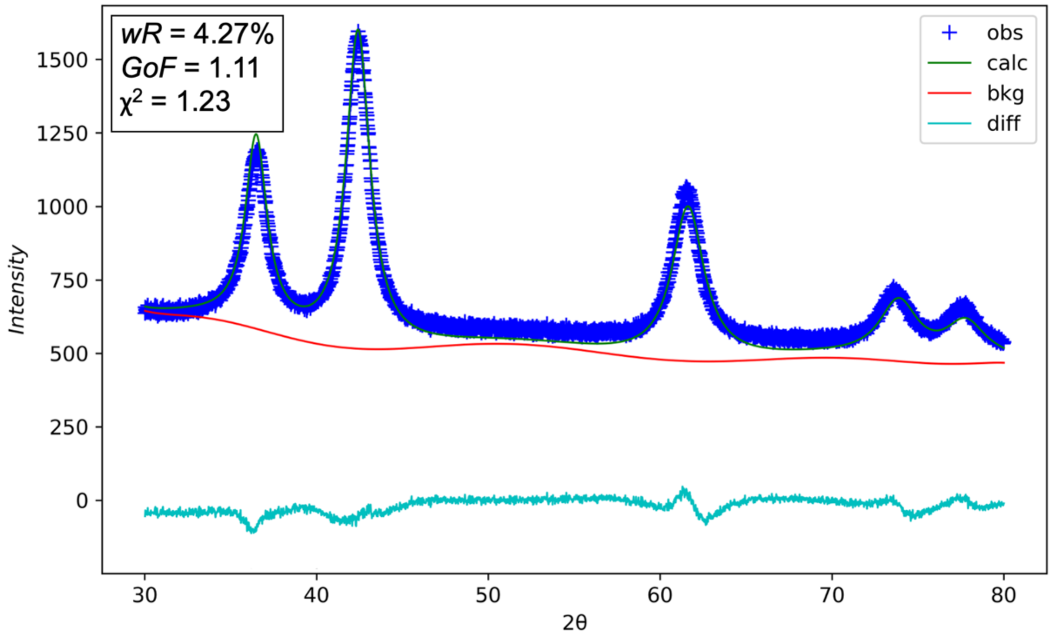

Figure S17. Powder XRD pattern of the optimized 6.6 nm CoO nanoparticles, with results from a Rietveld refinement to the $Fm\overline{3}m$ structure of rock salt CoO.

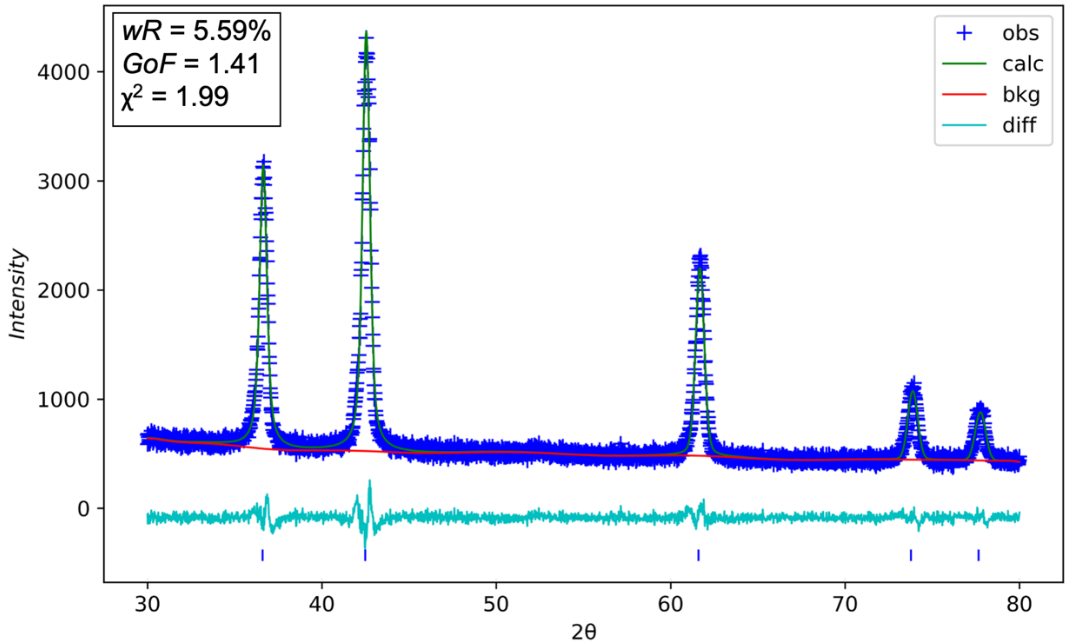

Figure S18. Powder XRD pattern of the unoptimized 65 nm CoO nanoparticles, with results from a Rietveld refinement to the $Fm\overline{3}m$ structure of rock salt CoO.

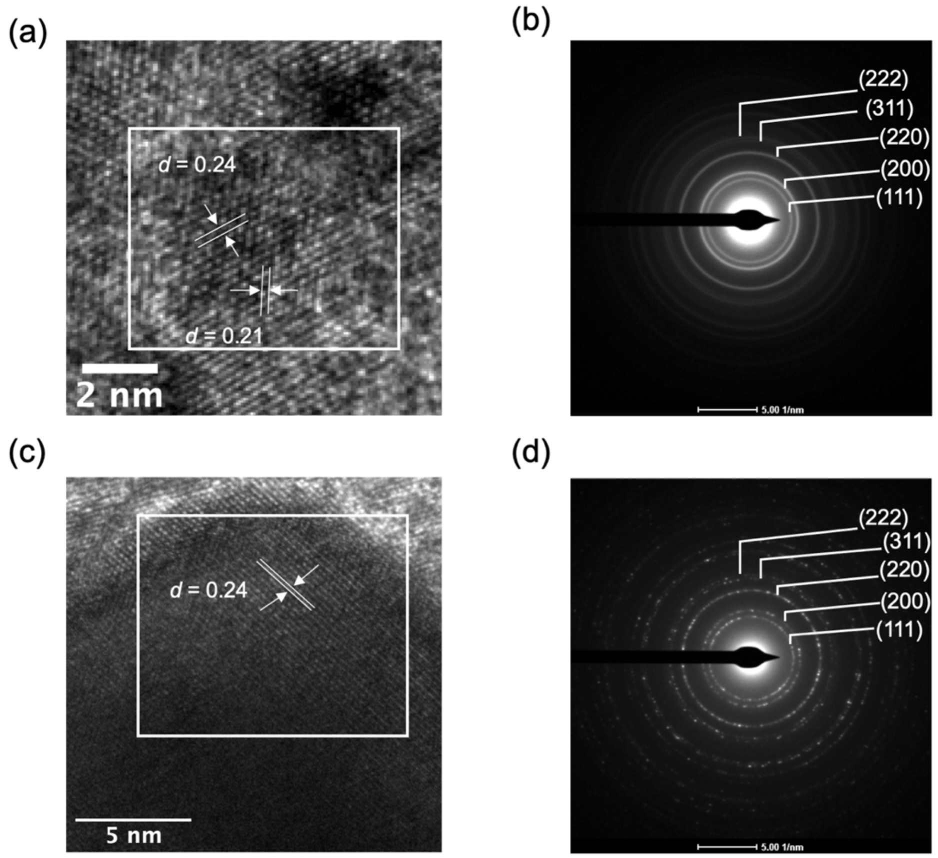

Figure S19. (a) HR-TEM and (b) SAED for the optimized 6.6 nm CoO nanoparticles. (c) HR-TEM and (d) SAED for the unoptimized, 65 nm CoO nanoparticles.

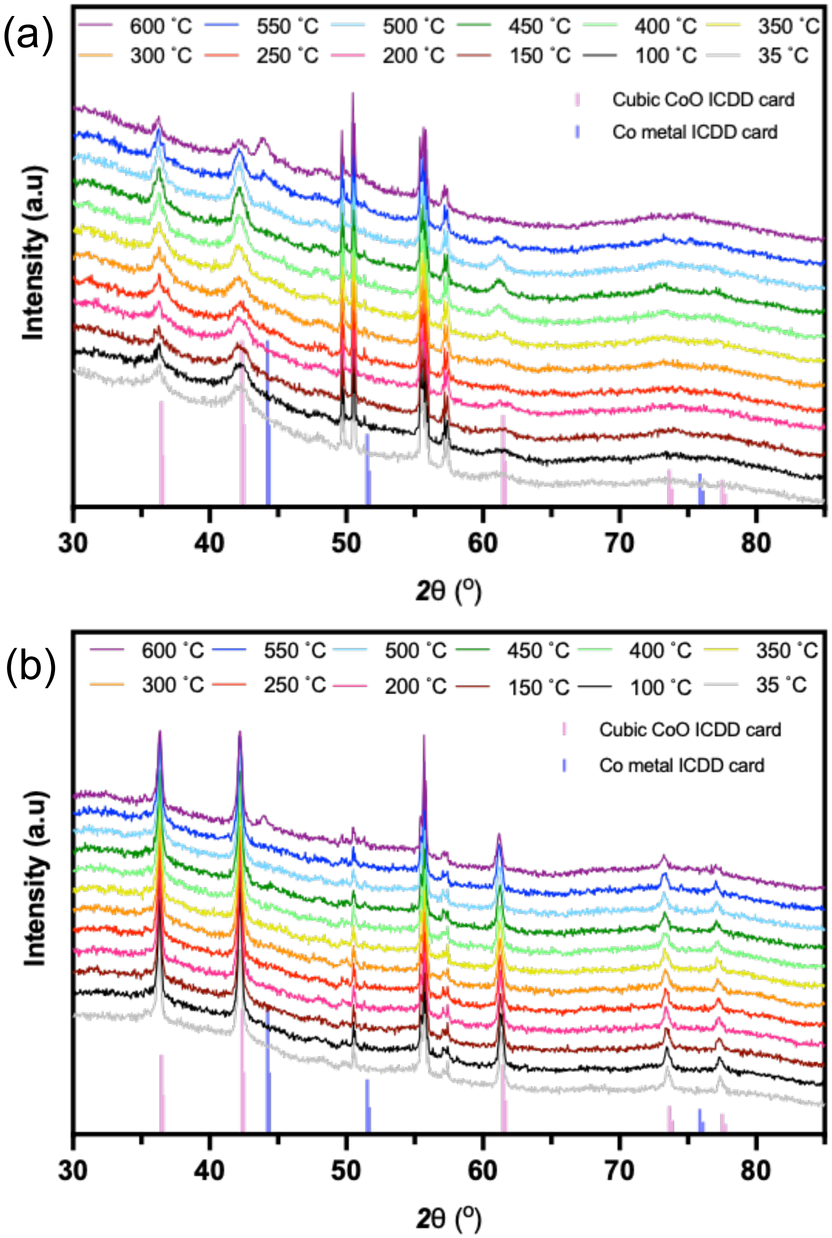

Figure S20. In situ powder XRD patterns collected from 35-600 °C in flowing 5% H_2 for (a) Opt-CoO/SiO_2, (b) Unopt-CoO/SiO_2, and (c) IWI-CoO/SiO_2. Diffraction peaks in (a) and (b) at 50.5, 55.6, 57.2° 2θ are associated with the quartz sample holder. The quartz sample holder was subtracted in (c). Stick patterns from ICDD reference cards for rock salt CoO and fcc Co metal, as well as spinel Co_3O_4 for the IWI-CoO/SiO_2 sample, are plotted below for comparison. (con't)

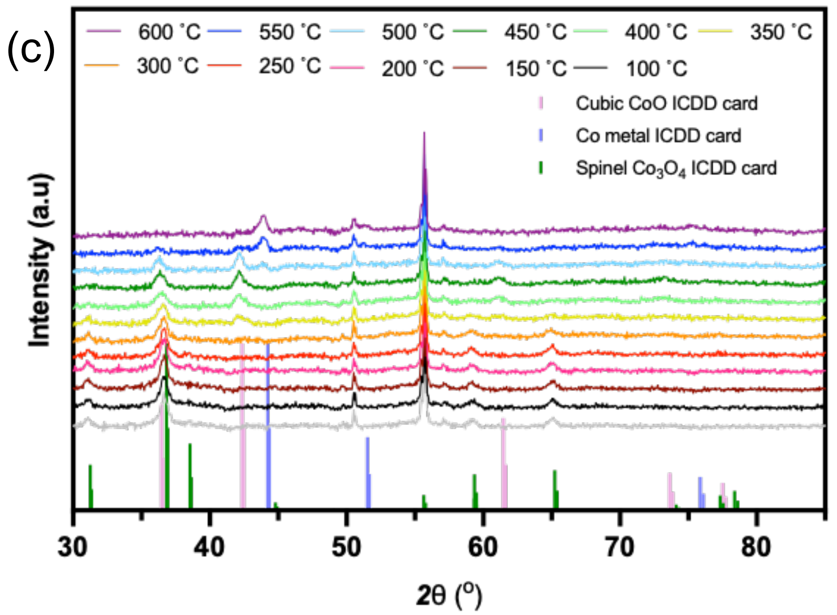

(con't) Figure S20. In situ powder XRD patterns collected from 35-600 °C in flowing 5% H_2 for (a) Opt-CoO/SiO_2, (b) Unopt-CoO/SiO_2, and (c) IWI-CoO/SiO_2. Diffraction peaks in (a) and (b) at 50.5, 55.6, 57.2° 2θ are associated with the quartz sample holder. The quartz sample holder was subtracted in (c). Stick patterns from ICDD reference cards for rock salt CoO and fcc Co metal, as well as spinel Co_3O_4 for the IWI-CoO/SiO_2 sample, are plotted below for comparison.

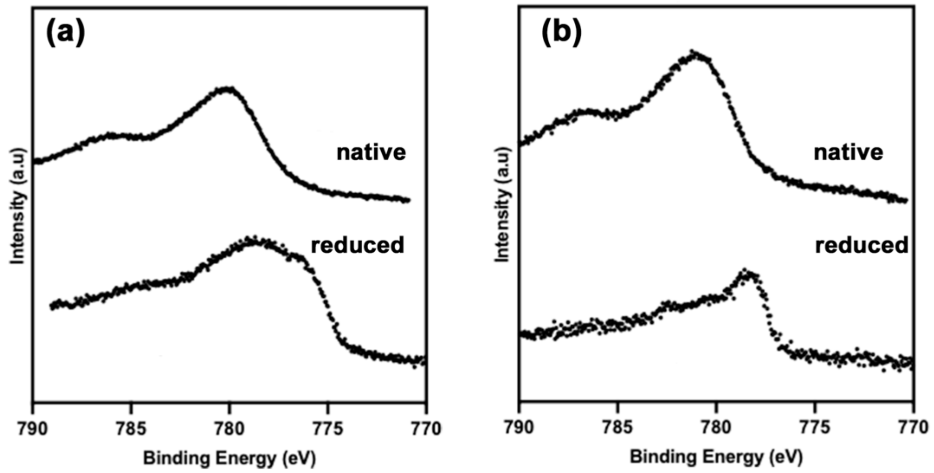

Figure S21. XPS spectra of the Co $2p_{3/2}$ region for (a) Opt-CoO/SiO_2 and (b) Unopt-CoO/SiO_2 catalysts in the as-synthesized forms (native) and following reduction at 450 °C.

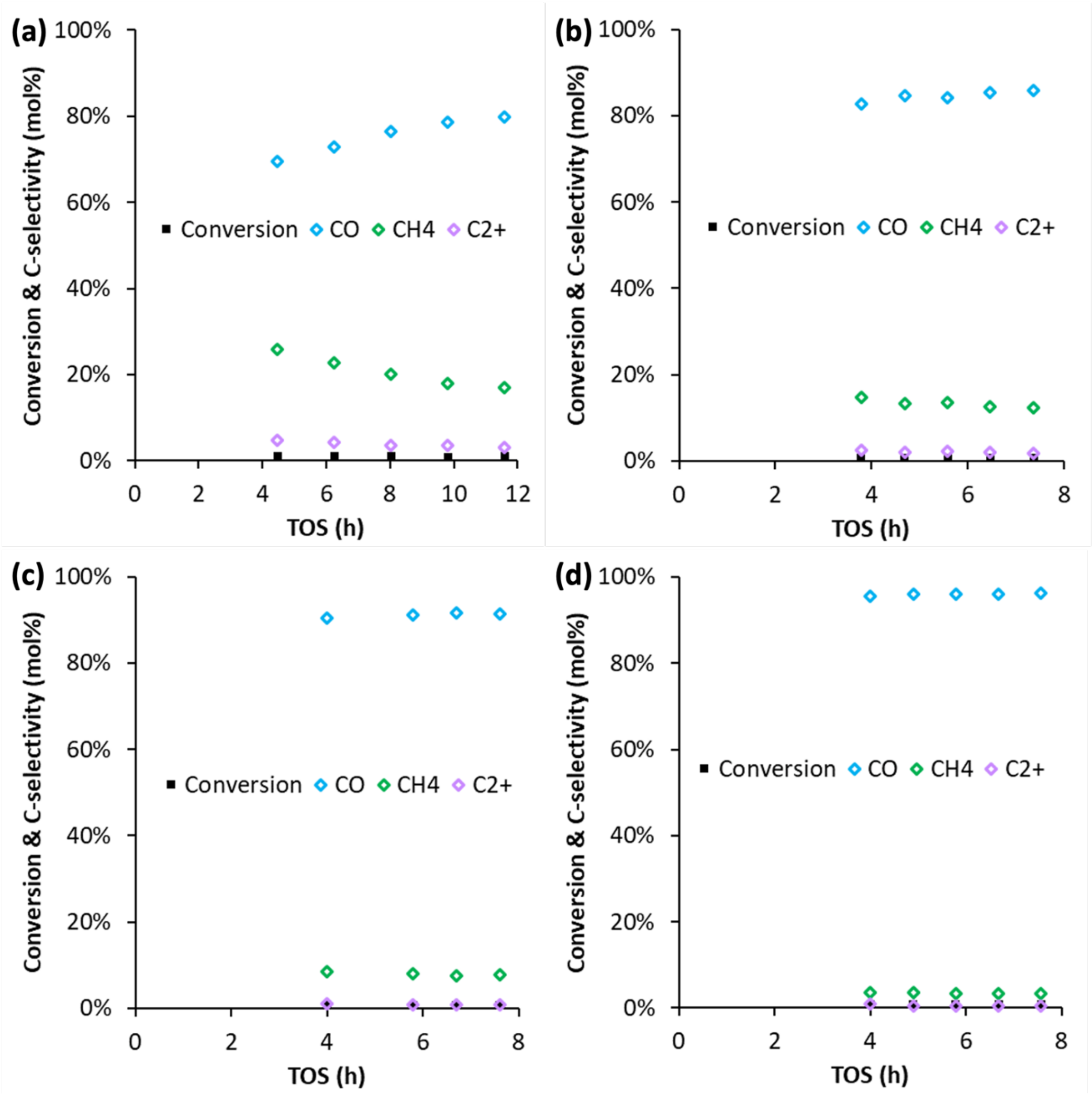

Figure S22. Conversion and product selectivity with time-on-stream (TOS) for CO_2 hydrogenation reaction over the Unopt-CoO/SiO_2 catalyst after reductive pretreatments at (a) 300, (b) 380, (c) 400, and (d) 450 °C. Reaction conditions were 300 °C, 3 MPa, WHSV of 1 g_{CO2}•g_{cat}^{-1}•h^{-1} and H_2:CO_2 molar ratio of 3. Data during the induction period at early TOS for each condition were not recorded.

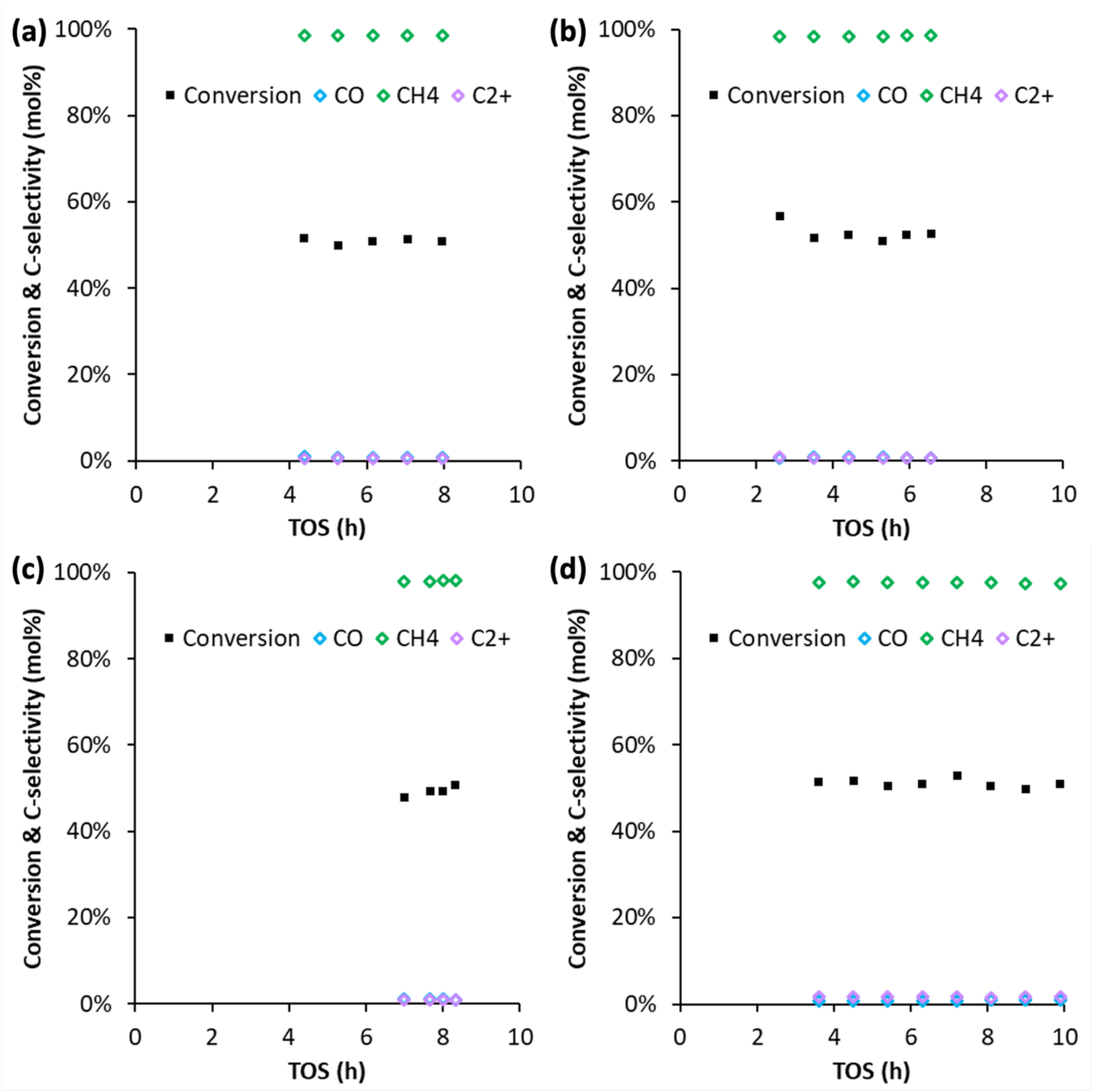

Figure S23. Conversion and product selectivity with time-on-stream (TOS) for CO_2 hydrogenation reaction over the Opt-CoO/SiO_2 catalyst after reductive pretreatments at (a) 300, (b) 380, (c) 400, and (d) 450 °C. Reaction conditions were 300 °C, 3 MPa, WHSV of 1 g_{CO2}•g_{cat}^{-1}•h^{-1} and H_2:CO_2 molar ratio of 3. Data during the induction period at early TOS for each condition were not recorded.

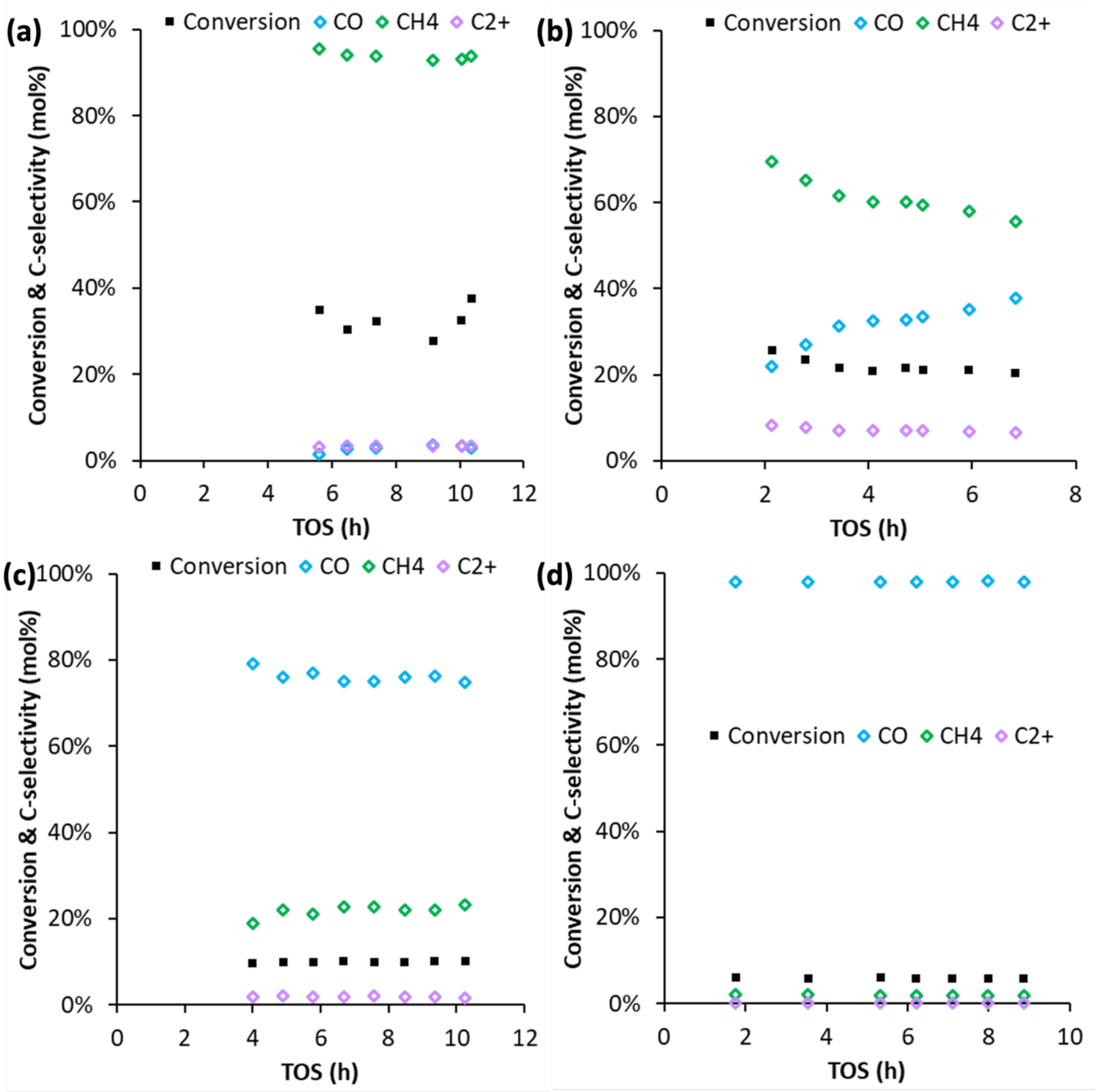

Figure S24. Conversion and product selectivity with time-on-stream (TOS) for CO_2 hydrogenation reaction over the IWI-CoO/SiO_2 catalyst after reductive pretreatments at (a) 300, (b) 380, (c) 400, and (d) 450 °C. Reaction conditions were 300 °C, 3 MPa, WHSV of 1 g_{CO2}•g_{cat}^{-1}•h^{-1} and H_2:CO_2 molar ratio of 3. Data during the induction period at early TOS for each condition were not recorded.

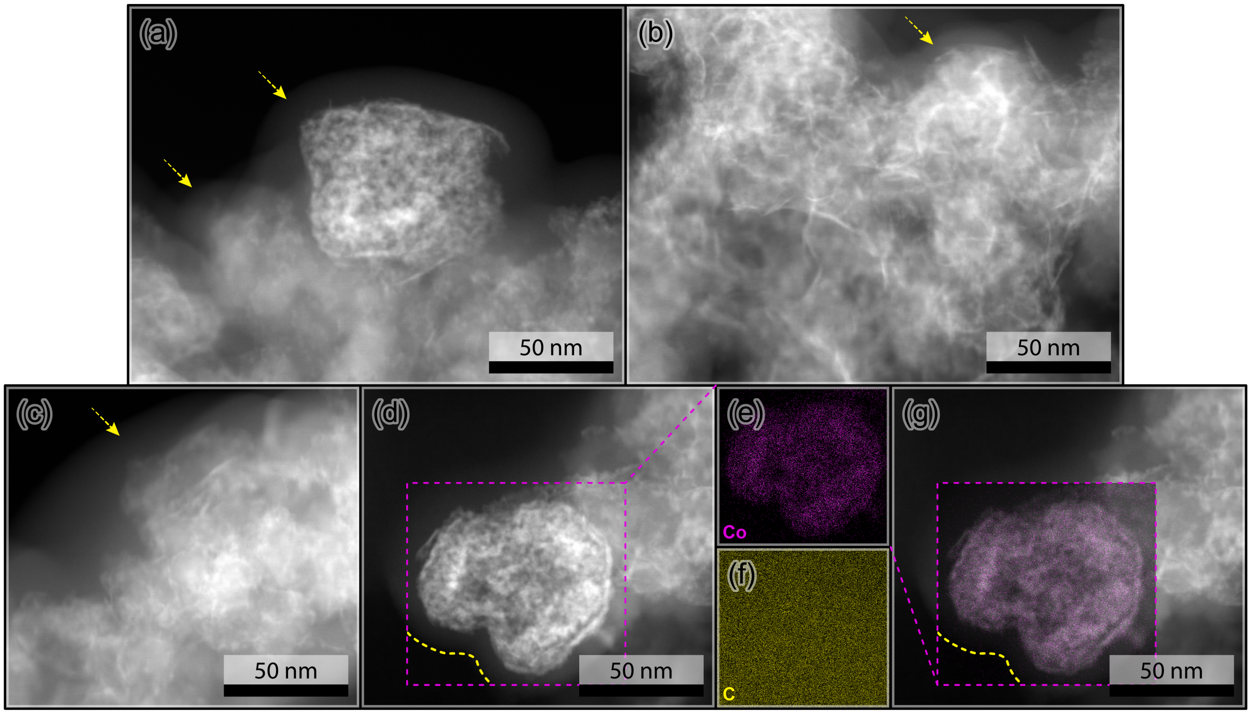

Figure S25. (a-d) HAADF-STEM images of cobalt-containing particles in the reduced (450 °C) Unopt-CoO/SiO_2 catalyst, showing a halo of carbon surrounding the particles, as indicated by yellow arrows. (e-f) STEM-EDS maps of Co and C, respectively, shown for the area indicated by the purple dotted box in (d), and overlaid on the same image in (g). The edge of the carbon shell is denoted with a yellow dotted line in (d) and (g), and EDS indicates a lack of other (non-carbon) elements in the shell region.

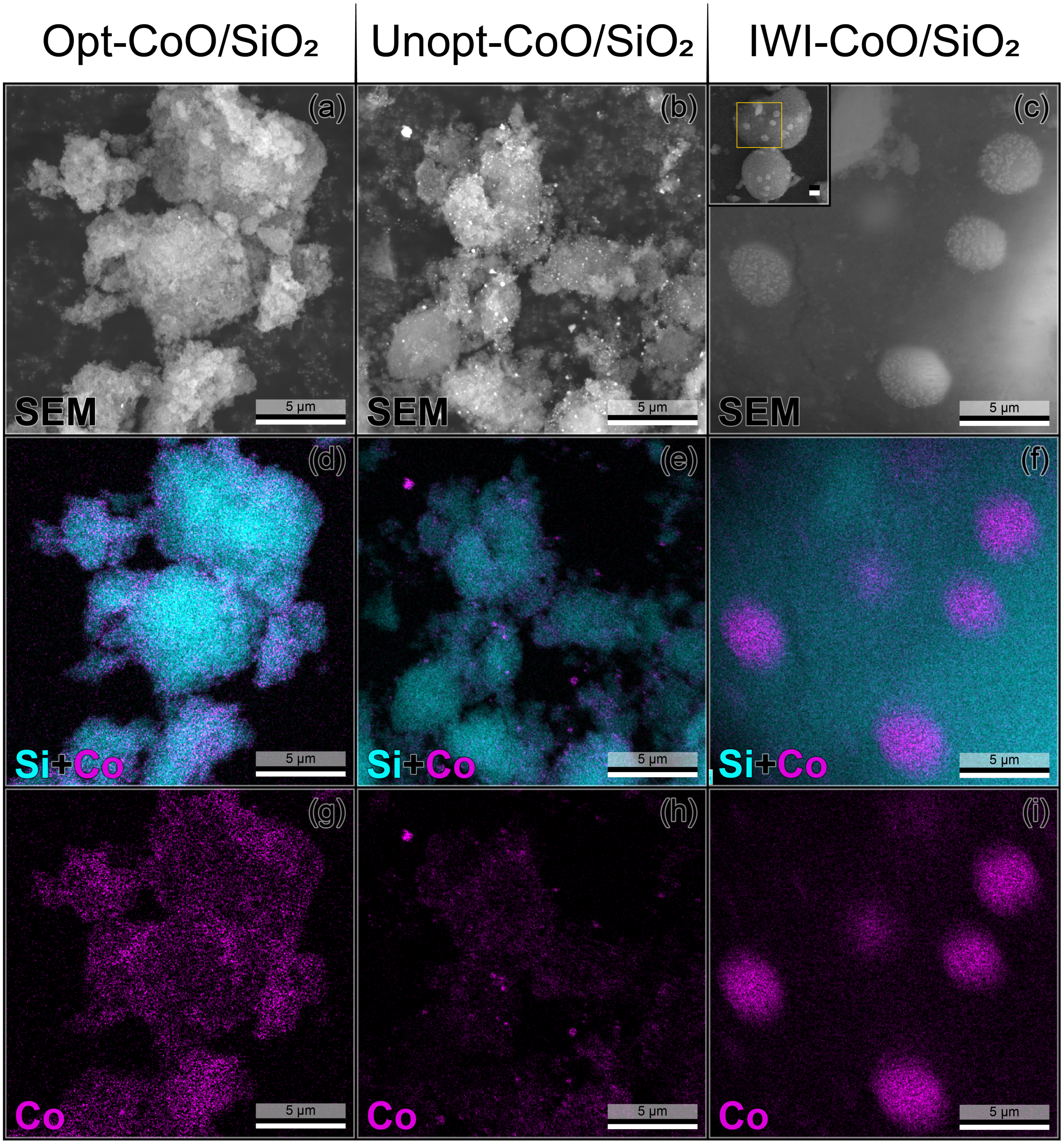

Figure S26. SEM images and EDS elemental maps of post-reaction (a, d, g) Opt-CoO/SiO_2, (b, e, h) Unopt-CoO/SiO_2, and (c, f, i) IWI-CoO/SiO_2 catalysts. Color indicates intensity for elemental maps (cyan for Si; magenta for Co). The inset in the IWI-CoO/SiO_2 SEM image indicates the region where the image and EDS maps were collected (yellow box), and the inset scale bar is 5 µm.

4. Code

Code for all previous sections can be found at: https://github.com/EmilyWill330

5. References

(1) Williamson, E. M.; Ghrist, A. M.; Karadaghi, L. R.; Smock, S. R.; Barim, G.; Brutchey, R. L. Creating Ground Truth for Nanocrystal Morphology: A Fully Automated Pipeline for Unbiased Transmission Electron Microscopy Analysis. *Nanoscale* **2022**. https://doi.org/10.1039/D2NR04292D.

(2) Williamson, E. M.; Sun, Z.; Mora-Tamez, L.; Brutchey, R. L. Design of Experiments for Nanocrystal Syntheses: A How-To Guide for Proper Implementation. *Chem. Mater.* **2022**, *34* (22), 9823–9835. https://doi.org/10.1021/acs.chemmater.2c02924.

(3) Shim, J. H.; Nam, K. M.; Seo, W. S.; Song, H.; Park, J. T. The Role of Water for the Phase-Selective Preparation of Hexagonal and Cubic Cobalt Oxide Nanoparticles. *Chem. - Asian J.* **2011**, *6* (6), 1575–1581. https://doi.org/10.1002/asia.201100031.

(4) Zhang, H.-T.; Chen, X.-H. Controlled Synthesis and Anomalous Magnetic Properties of Relatively Monodisperse CoO Nanocrystals. *Nanotechnology* **2005**, *16* (10), 2288–2294. https://doi.org/10.1088/0957-4484/16/10/051.

(5) Wang, H.; Si, H.; Zhao, H.; Du, Z.; Li, L. S. Shape-Controlled Synthesis of Cobalt Oxide Nanocrystals Using Cobalt Acetylacetonate. *Mater. Lett.* **2010**, *64* (3), 408–410. https://doi.org/10.1016/j.matlet.2009.11.034.

(6) Si, H.; Wang, H.; Shen, H.; Zhou, C.; Li, S.; Lou, S.; Xu, W.; Du, Z.; Li, L. S. Controlled Synthesis of Monodisperse Manganese Oxide Nanocrystals. *CrystEngComm* **2009**, *11* (6), 1128. https://doi.org/10.1039/b901603c.

(7) Saddeler, S.; Hagemann, U.; Schulz, S. Effect of the Size and Shape on the Electrocatalytic Activity of Co_3O_4 Nanoparticles in the Oxygen Evolution Reaction. *Inorg. Chem.* **2020**, *59* (14), 10013–10024. https://doi.org/10.1021/acs.inorgchem.0c01180.

(8) Wong, T.-T. Performance Evaluation of Classification Algorithms by K-Fold and Leave-One-out Cross Validation. *Pattern Recognit.* **2015**, *48* (9), 2839–2846. https://doi.org/10.1016/j.patcog.2015.03.009.

(9) Frey, D.; Shin, J. H.; Musco, C.; Modestino, M. A. Chemically-Informed Data-Driven Optimization (ChIDDO): Leveraging Physical Models and Bayesian Learning to Accelerate Chemical Research. *React. Chem. Eng.* **2022**, *7* (4), 855–865. https://doi.org/10.1039/D2RE00005A.

(10) Cardoso, T. N. C.; Silva, R. M.; Canuto, S.; Moro, M. M.; Gonçalves, M. A. Ranked Batch-Mode Active Learning. *Inf. Sci.* **2017**, *379*, 313–337. https://doi.org/10.1016/j.ins.2016.10.037.
